# Identifying the key genes of Epstein–Barr virus‐regulated tumour immune microenvironment of gastric carcinomas

**DOI:** 10.1111/cpr.13373

**Published:** 2022-12-14

**Authors:** Heng Zhou, Shuili Jing, Yu Liu, Xuming Wang, Xingxiang Duan, Wei Xiong, Ruohan Li, Youjian Peng, Yilong Ai, Dehao Fu, Hui Wang, Yaoqi Zhu, Zhi Zeng, Yan He, Qingsong Ye

**Affiliations:** ^1^ Center of Regenerative Medicine & Department of Stomatology Renmin Hospital of Wuhan University Wuhan China; ^2^ College of Life and Health Sciences, Institute of Biology and Medicine Wuhan University of Science and Technology Wuhan Hubei China; ^3^ Department of Pathology Guilin Medical University Guilin Guangxi China; ^4^ Foshan Hospital of Stomatology, School of Medicine, Foshan University Foshan Guangdong China; ^5^ Department of Orthopaedics, Shanghai General Hospital Shanghai Jiao Tong University School of Medicine Shanghai China; ^6^ Demonstration Center for Experimental Basic Medicine Education, Wuhan University Wuhan China; ^7^ Institute of Regenerative and Translational Medicine Tianyou Hospital of Wuhan University of Science and Technology Wuhan Hubei China; ^8^ Department of oral and maxillofacial surgery Hospital of Taikang Tongji (Wuhan) Wuhan China; ^9^ Department of Pathology Renmin Hospital of Wuhan University Wuhan China; ^10^ Department of oral and maxillofacial surgery, Massachusetts General Hospital Harvard Medical School Boston Massachusetts USA

## Abstract

The Epstein–Barr virus (EBV) is involved in the carcinogenesis of gastric cancer (GC) upon infection of normal cell and induces a highly variable composition of the tumour microenvironment (TME). However, systematic bioinformatics analysis of key genes associated with EBV regulation of immune infiltration is still lacking. In the present study, the TCGA and GEO databases were recruited to analyse the association between EBV infection and the profile of immune infiltration in GC. The weighted gene co‐expression analysis (WGCNA) was applied to shed light on the key gene modules associated with EBV‐associated immune infiltration in GC. 204 GC tissues were used to analysed the expression of key hub genes by using the immunohistochemical method. Real‐time PCR was used to evaluate the association between the expression of EBV latent/lytic genes and key immune infiltration genes. Our results suggested that EBV infection changed the TME of GC mainly regulates the TIICs. The top three hub genes of blue (GBP1, IRF1, and LAP3) and brown (BIN2, ITGAL, and LILRB1) modules as representative genes were associated with EBV infection and GC immune infiltration. Furthermore, EBV‐encoded LMP1 expression is account for the overexpression of GBP1 and IRF1. EBV infection significantly changes the TME of GC, and the activation of key immune genes was more dependent on the invasiveness of the whole EBV virion instead of single EBV latent/lytic gene expression.

## Graphical abstract

The lytic EBV virion infects host cells, activates the immune response of the host and promotes the expression of associated key hub genes. Then EBV establishes latent infection in host cells and expressed LMP1, which promotes activation of the NF‐κB signalling pathway and expression of GBP1 and IRF1.

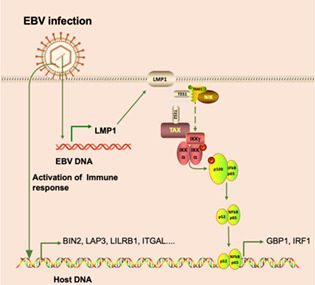

## 1 INTRODUCTION

Epstein–Barr virus (EBV) infects about 90% of the adult population, and establishes latent infection in healthy people, but does not cause major symptoms in most lifelong hosts.^1^, ^2^, ^3^ Nevertheless, EBV has a strong carcinogenic ability and has been described to be the nosogenesis of several malignances, such as B cell or NK‐T cell lymphoma, also to be associated with epithelial cancers, such as nasopharyngeal carcinoma (NPC) and more recently to gastric cancer (GC).^4^, ^5^

GC is the fifth most common cancer and the second leading cause of cancer‐related death.^6^, ^7^ Carcinogenesis of GC involves multistep processes, in which EBV seems to be correlated with the mechanism of a few cases.^8^, ^9^ Studies have indicated that EBV infection can be detected in nearly 10% of GC lesion, which lead the scientific community to explore the role of EBV in the pathological process of GC.^10^, ^11^ Importantly, the Cancer Genome Atlas (TCGA) has classified EBV‐positive GC (EBVaGC) as one of four subtypes.^12^, ^13^ EBVaGC has some different characteristics, such as PD‐L1 amplification,^14^ frequent mutations of PI3KCA,^15^ altered DNA methylation profiles.^16^ In addition, EBVaGC has been certificated to come from monoclonal amplification of EBV‐infected normal gastric cells, which demonstrates that EBV infection may involve in early carcinogenesis.^17^

In an immunocompetent host, both innate and adaptive immune responses are induced by EBV infection, resulting in the suppression of viral replication. In addition to the possession of tumour cell‐transforming properties, EBV can also affect the properties and compositions of the cells present in the tumour microenvironment (TME).^18^ Non‐transformed cells compose the major immune cells of TME, which lead to the reactive and inflammatory appearance of TME.^19^ TME includes cytokines, chemokines, and other bioactive matters. In addition, the specific proteins expressed during EBV lytic and latent infection lead to activation of the CD4+, CD8+ T cells, and natural killer (NK) cells to induce antiviral immunity.^20^ Importantly, the TME is critically important in disease pathogenesis, as the tumour cells need to evade anti‐EBV immune responses and, in many instances, even require support from TME cells.^21^ Hence, it is important to identify the essential genes that regulate the TME of EBVaGC.

In this study, the TCGA and GEO databases were recruited to analyse the association between EBV infection and the profile of immune infiltration in GC. The weighted gene co‐expression analysis (WGCNA) was applied to shed light on the keg gene modules associated with EBV‐associated immune infiltration in GC. GC tissue microarrays were used to analysed the expression of key hub genes between EBV‐ and EBV+ tumour samples. Furthermore, we found that LMP1 expression is account for the overexpression of GBP1 and IRF1. Importantly, the activation of key immune genes was more dependent on the invasiveness of the whole EBV virion instead of single EBV latent/lytic gene expression.

## 2 METHODS AND MATERIALS

### 2.1 Data collection

To evaluate the role of EBV in immune‐regulated function in GC, gene expression profiling for EBV‐associated tumour samples was obtained from the TCGA‐STAD project (*n* = 265).^8^ The corresponding clinical profiles were also downloaded from the TCGA portal as described previously.^22^ GSE66229 includes 300 gastric tumour samples with EBV infection status detected by the FISH method, as well as GSE51575 includes 52 samples. The mRNA data of GSE66229^23^ and GSE51575^24^ were normalized using the RMA method.

### 2.2 Evaluating the immune infiltration profiles changes by EBV infection

To analyse the immune infiltration profiles changes by EBV infection in GC, the TCGA‐STAD samples were divided into two groups according to EBV infection status. The algorithm ESTIMATE provides stromal, immune, and ESTIMATE scores by performing ‘limma’ and ‘estimate’ with TCGA‐STAD data.^25^ To estimate the definitive proportions of two types of stromal cells and eight types of immune cells in the EBV‐associated samples, the R package (microenvironment cell populations‐counter, MCP‐counter) was used to quantify the proportion of cells in individual tissues from gene expression, and export them. In addition, the absolute‐mode CIBERSORT algorithm was performed based on the LM22 gene signature. Gene expression data of TCGA‐STAD were prepared using standard annotation files and evaluated using CIBERSORT (http://ciber sort.stanford.edu/), with the algorithm run at 1000×.

### 2.3 Differentially expressed genes between distinct EBV infection status

Differentially expressed genes (DEGs) were analysed by using the ‘limma’ package in R language with the standard comparison model. The adjusted *p* values for multiple testing were obtained from an embedded Benjamini–Hochberg procedure. Details on cutoffs were as follows: fold change >1 or < −1 and adj. *p* < 0.05. Volcano plots were obtained using the visual tool ‘Hiplot.’ To perform gene ontology (Go) and Kyoto Encyclopedia of Genes and Genomes (KEGG) enrichment analysis, the DEGs from the intersection part of three datasets were input in R with ‘clusterProfiler’ and ‘org.Hs.eg.db,” and the significance threshold was *p* ≤ 0.05.

### 2.4 WGCNA network analysis

The WGCNA analysis was performed in R and used to build a gene co‐expression network to mine their module membership associated with associated immune cell distribution of corporate DEGs. To identify the modules that were most strongly related to immune cell distribution, the samples were divided into two groups according to MCP count and ESTIMATE score. The cutoff value was set as 50%. To obtain co‐expressed modules, the minimum number of genes in the module was 20, the merging threshold of similar modules was 0.2, and the default parameters were used for the rest. Then the topological overlap matrix (TOM) is constructed by using block modular functions. The dynamic tree‐cutting algorithm is used for clustering. Genes with similar expression patterns were grouped into the same co‐expression module, and different colours were assigned to each co‐expression module for discrimination. Finally, gene significance (GS) and module membership (MM) were calculated, and modules were correlated with immune cell distribution and score. Genes in the co‐expression module have high connectivity and genes in the same module may have similar biological functions.

### 2.5 Identification and validation of hub genes

Hub genes in each co‐expressed module were defined according to the Eigengene‐based module connectivity or module membership (*k*_ME_) index in non‐preserved modules. To determine the *k*_ME_, the correlation of the expression value of a gene and eigengene of the module was estimated. This index measures the closeness of a gene in a given module. Genes with *k*_ME_ ≥0.7 were considered as hub genes in the respective module. The top three genes of each module were collected for further analysis.

### 2.6 Identification of immune‐associated genes in EBVaGC

The immune‐related genes in DEGs of EBVaGC were identified using the Immport (https://www.immport.org/shared/genelists), which provides a gene list including 1795 immune‐related genes. The protein–protein interaction (PPI) network was constructed by using cytoscape 3.7.2 on Mac OS.

### 2.7 The prognosis evaluation of COPS subunits in HNSCC

The Kaplan–Meier plotter (www.kmplot.com) provides the prognostic analysis of 54,000 genes in 21 cancer types within GEO, EGA and TCGA datasets. The association between mRNA expression of key genes and the prognosis of GC was analysed using the mRNA Chip module.^26^ To perform the analysis of the survival events, all patients were divided into two groups automatically on the basis of computerized optimal outcomes. The hazard ratio (HR) with 95% confidence intervals and log‐rank *p*‐value were obtained to evaluate the prognostic difference.

### 2.8 Assessment of correlation of key genes with tumour immune infiltration

Tumour Immune Estimation Resource (TIMER) (https://cistrome.shinyapps.io/timer/) provides immune infiltration information containing each tumour sample's immune cell fraction using various algorithms.^27^ The correlation of tumour immune cell infiltrating in TCGA‐STAD with key genes was analysed to explore whether the risk signature could act as a novel and reliable indicator in the TME of GC. To assess the relationship between the expression profiles of key genes and cell subsets, single‐cell sequencing was used. An online single‐cell sequencing analysis tool (http://tisch.comp-genomics.org/home/) with GSE134520 and GSE167297 datasets was applied.

### 2.9 Gene set enrichment analysis

Gene set enrichment analysis (GSEA) was conducted for key genes on the GSEA portal website (http://www.broad.mit.edu/GSEA/) with the following parameter settings: phenotype = 35% high expression versus 35% low expression; permutation: sample, permutations = 1000. The gene set size was 15 < *n* < 500; probe set collapse = false; annotation = HALLMARK. The TCGA‐STAD dataset was included. The GSEA results were separately shown based on the pathway function. FDR <0.25, *p* < 0.05 and NES value >1 were considered as significant.

### 2.10 The PPI networks construction

The topological features of PPI networks were used in analysing the functional modules in networks. The Molecular Complex Detection (MCODE) algorithm was used to analysis the association of various key module genes.^28^ The PPI correlation was obtained from STRING (https://cn.string-db.org) and the pictures were drawn with cytoscape 3.72. To perform the functional analysis of hub genes, a web tool Metascape (https://metascape.org/gp/index.html#/main/step1) was used, which is a powerful tool for gene function annotation and analysis. It can help users to apply the currently popular bioinformatics analysis methods to batch gene and protein analysis, to realize the knowledge of gene or protein function.

### 2.11 Cell culture

One human GC cell line, MGC‐803, was cultured in Dulbecco's Modified Eagle Medium (Hyclone) containing 10% fetal bovine serum (Gibco) in a humidified atmosphere at 37°C, containing 5% CO_2_.

### 2.12 Cell transfection

The pSG5‐EBNA1 and pSG5‐LMP1 were described in our previous works. The pSG5‐BMRF1 and pSG5‐BGLF4 were kindly provided by Prof. Xiaoping Sun (Wuhan University). pcDNA3.1‐LMP2A was a gift from Prof. Bin Luo (Qingdao University). 1 ×10^5^ MGC‐803 cells were seeded into 12‐well plates. After adhering, cells were transfected with plasmids using Neofect reagents (Mayin, Beijing, China) according to the protocol from manufacturers.

### 2.13 Tissue sample and immunohistochemistry

Totally, 204 cases of gastric were included in this study. The involved tumour patients were come from the department of pathology of Guilin Medical University and definitely diagnosed in the Department of Pathology during 2015–2017. The clinical sample studies were reviewed by the Ethics Research Committee of the Affiliated Hospital of Guangxi Guilin Medical College (GLMU1A201807). The EBV infection was detected with the in situ hybridization experiment for EBER. Part of the tumour tissue is extracted to make tissue microarrays. The expression of GBP1, BIN2, and LAP3 was detected by immunohistochemistry (IHC) assay. After sectioning, paraffin tissue microarray sections were used to dehydrate and repair. The microarrays were then quenched with 3% hydrogen peroxide and blocked with 10% goat serum for 30 min. Then, the sections were incubated overnight with primary antibody for GBP1 (Santa Cruz, sc‐53857, 1:50), BIN2 (Santa Cruz, sc‐376391, 1:50) and LAP3 (Santa Cruz, sc‐376270, 1:50) at 4 °C. The sections were washed with PBS for three times and incubated with a secondary antibody (Proteintech, China) for 30 min at indoor temperature. Subsequently, the tissue section was orderly stained with DAB and Harris haematoxylin. To evaluate IHC staining intensity, the IHC Toolbox plug‐in of ImageJ software was used to calculate grayscale values.

### 2.14 Quantitative real‐time PCR

For specific RT‐PCR steps, please refer to the previous publication. The primer sequences used in this study were as follows: GBP1 forward, 5′‐ACAACTCAGCTAACTTTGTGGG‐3′ and reverse 5′‐TGATACACAGGCGAGGCATATTA‐3′; IRF1 forward, 5′‐GCAGCTACACAGTTCCAGG‐3′ and reverse 5′‐GTCCTCAGGTAATTTCCCTTCCT‐3′; LAP3 forward, 5′‐TCGGCAAAGCTCTATGGAAGT‐3′ and reverse 5′‐GCGTCATCTCATTGGCTGG‐3′; BIN2 forward, 5′‐GGTCGGAAACTCGTGGACTAT‐3′ and reverse 5′‐GAAGACATCCCTCAAGTTGGAAA‐3′; ITGAL forward, 5′‐TGCTTATCATCATCACGGATGG‐3′ and reverse 5′‐CTCTCCTTGGTCTGAAAATGCT‐3′; LILRB1 forward, 5′‐CTGTTACTATGGTAGCGACACTG‐3′ and reverse 5′‐CACACTGGAGGATTACATTCCC‐3′; glyceraldehyde‐3‐phosphate dehydrogenase (GAPDH) forward, 5′‐CAGGAGGCATTGCTGATGAT‐3′ and reverse 5′‐GAAGGCTGGGGCTCATTT‐3′. The associated gene expression was calculated by normalizing it to GAPDH by using a △△GA method.

### 2.15 Statistical analysis

The HR with a *p*‐value was applied to determine the significance of overall survival (OS). Student's *t*‐test was applied to evaluate the significance between the two groups. Spearman's correction was used to assess the association of key hub genes and the strength of the correlation. The Student's *t*‐test was used to analyse the experimental data. The results were considered statistically significance as *p < 0.05, **p < 0.01, ***p < 0.001 and ****p < 0.0001.

## 3 RESULTS

### 3.1 EBV infection alters the profiles of immune infiltration in GC

**FIGURE 1:**
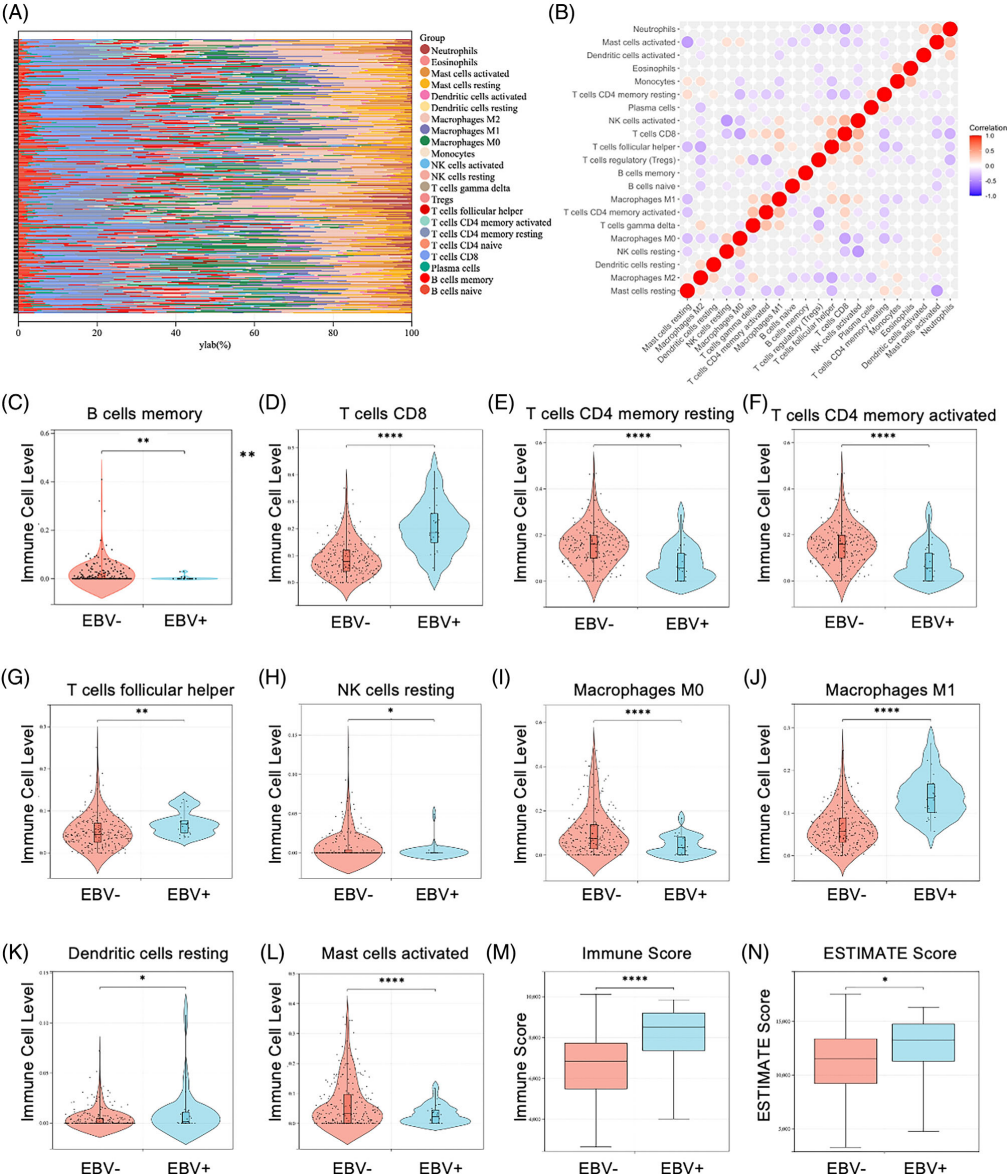
The analysis of immune infiltration of gastric cancer (GC) with EBV *infection*. Heatmap showed the distribution state in the EBV+ and EBV‐ GC tissue samples (A). The correlation among tumour‐infiltrating immune cells (TIICs) in the EBV+ and EBV‐ GC tissue samples (B). Distributions and significances of 21 TIICs were analysed by CIBERSORT algorithm. The correlation among TIICs in the EBV‐negative and EBV‐positive tissue samples (C)–(L). Total 10 TIICs presented a significant difference in the distribution between EBV‐negative and EBV‐positive tissue tumour samples. The ESTIMATE algorithm was used to calculate the immune (M) and ESTIMATE scores (N) from the TCGA‐SWAD dataset. *p < 0.05; **p < 0.01; ***p < 0.001; ****p < 0.0001.

To determine whether EBV infection affects the proportion of tumour‐infiltrating immune cells (TIICs) in GC, the CIBERSORT algorithm was adopted. Interestingly, the distribution of TIICs varied based on the kinds of cells. Some TIICs had more immune cells in EBVaGC tumour tissues, whereas other TIICs showed the less ratios in EBV‐tumour tissues (Figure 1A). No significant correlation was found between EBV‐ and EBV+ tissues with regard to numerical values (Figure 1B). A landscape was presented to detect the distribution and salience of TIICs (Figure S1B). Compared with EBV‐ samples, EBVaGC tissues had more proportions of CD8+ T cells (Figure 1D), T cells follicular helper (Figure 1G) and macrophage M1 (Figure 1J), as well as less ratio of B cell memory (Figure 1C), T cell CD4 memory resting (Figure 1E), NK cells resting (Figure 1H), T cell CD4 memory activated (Figure 1F), macrophage M0 (Figure 1I), dendritic cells (DCs) resting (Figure 1K) and mast cells activated (Figure 1L). In addition, we employed another immune infiltrate method‐MCP‐counter to reanalyze the data. Results showed that EBV infection increased the proportions of T cells, cytotoxic lymphocytes, CD8+ T cells, NK cells, monocyticlineage and myeloid DCs (Figure S1A). In EBV‐negative tissues, neutrophils and fibroblasts have more ratios (Figure S1A). To further evaluate whether EBV infection was associated with immune infiltration of GC, ESTIMATE scores were used to calculate the immune and stromal scores. We calculated the immune and stromal scores in the EBV‐negative (EBV‐) and EBV‐positive (EBV+) samples of TCGA‐SWAD, respectively. EBV infection significantly increased the immune (Figure 1M) and ESTIMATE scores (Figure 1N) compared with EBV‐negative tissues, whereas had no significant affection on the stromal score (Figure S1C). These results suggested that EBV alters the profile of immune infiltration and promotes inflammatory response in GC tissues.

### 3.2 Identification of DEGs in three gene datasets

**FIGURE 2:**
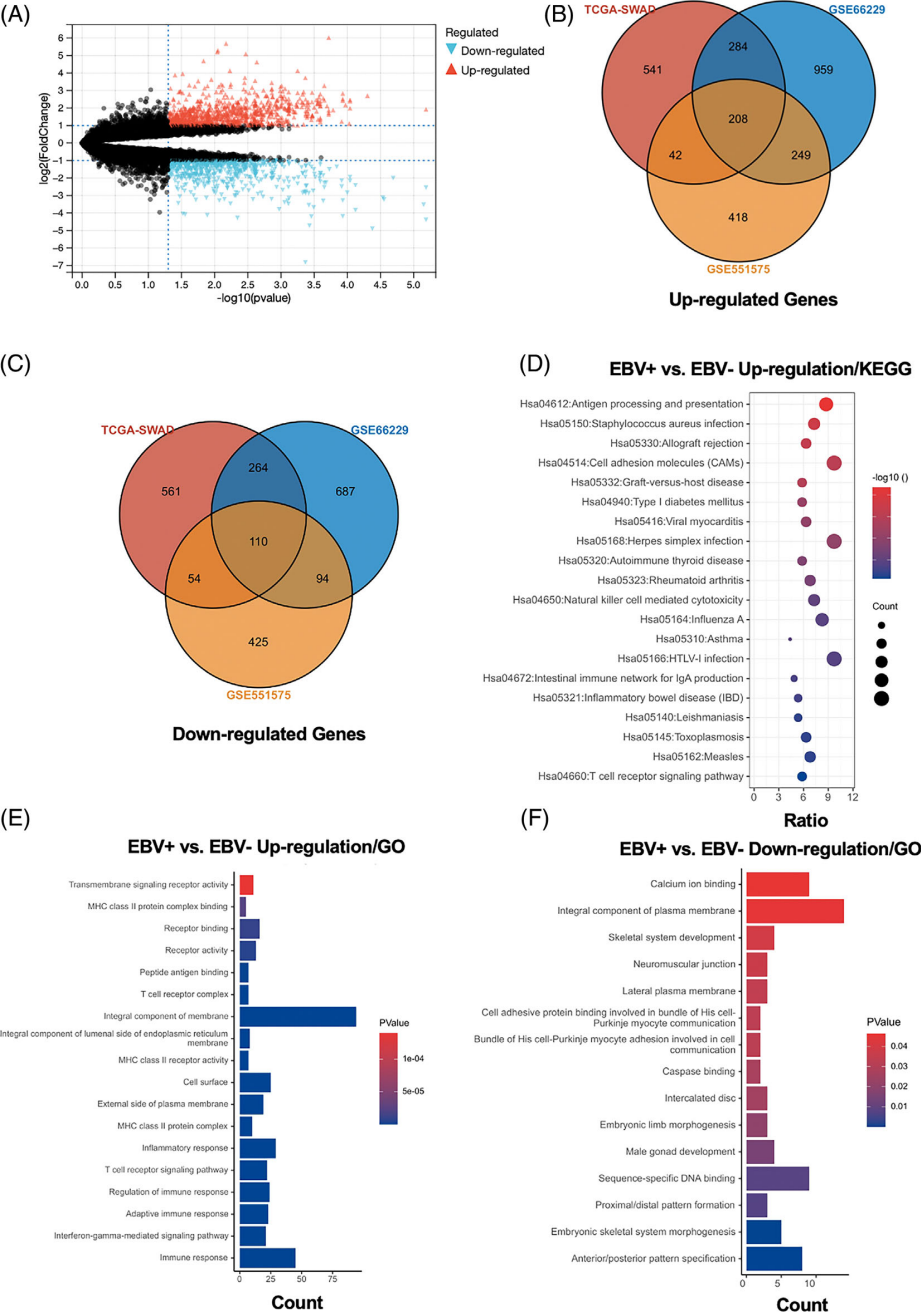
Identification of differentially expressed genes (DEGs) with EBV infection in gastric cancer. The volcano pictures showed the DEG distribution of GSE551575 (A). Venn diagram concluded the upregulated (B) and downregulated (C) genes between GSE551575, GSE66229 and TCGA‐SWAD datasets. KEGG pathway analyses of upregulated DEGs (D). GO enrichment analyses of upregulated (E) or downregulated differentially expressed genes (F)

To determine the expression profiles regulated by EBV infection, three gene datasets were included in the analysis. The volcano pictures showed the DEG distribution of GSE551575 (Figure 2A), GSE66229 (Figure S2A) and TCGA‐SWAD (Figure S2B). The three datasets included 208 same upregulated genes (Figure 2B, Table S1) and 110 same downregulated genes (Figure 2C and Table S2). Then, we performed GO and KEGG pathway enrichment analyses of upregulated or downregulated DEGs. The top five significant GO enrichment results of biological process (BP), cellular compound and molecular function, and 15 KEGG signalling pathways were presented. The up‐regulated GO analysis included immune response, interferon‐gamma‐mediated signalling pathway, adaptive immune response, regulation of immune response and T cell receptor signalling pathway et al., which are significantly associated with immune and inflammation response (Figure 2D and Table S3). The down‐regulated GO analysis included anterior/posterior pattern specification, embryonic skeletal system morphogenesis, proximal/distal pattern formation, male gonad development and embryonic limb morphogenesis et al. (Figure 2E and Table S4). The upregulated DEGs were mostly enriched in antigen processing and presentation, *Staphylococcus aureus* infection, allograft rejection, cell adhesion molecules and graft‐versus‐host disease et al. (Figure 2F and Table S5). However, only peroxisome pathways were associated with down‐regulated DEGs between EBV+ versus EBV‐ GC tissues (Figure S2C and Table S5). These results suggested that EBV infection was strongly associated with the activation of the immune response of GC tissues.

### 3.3 Identification of modules involved in the response to immune infiltration

**FIGURE 3:**
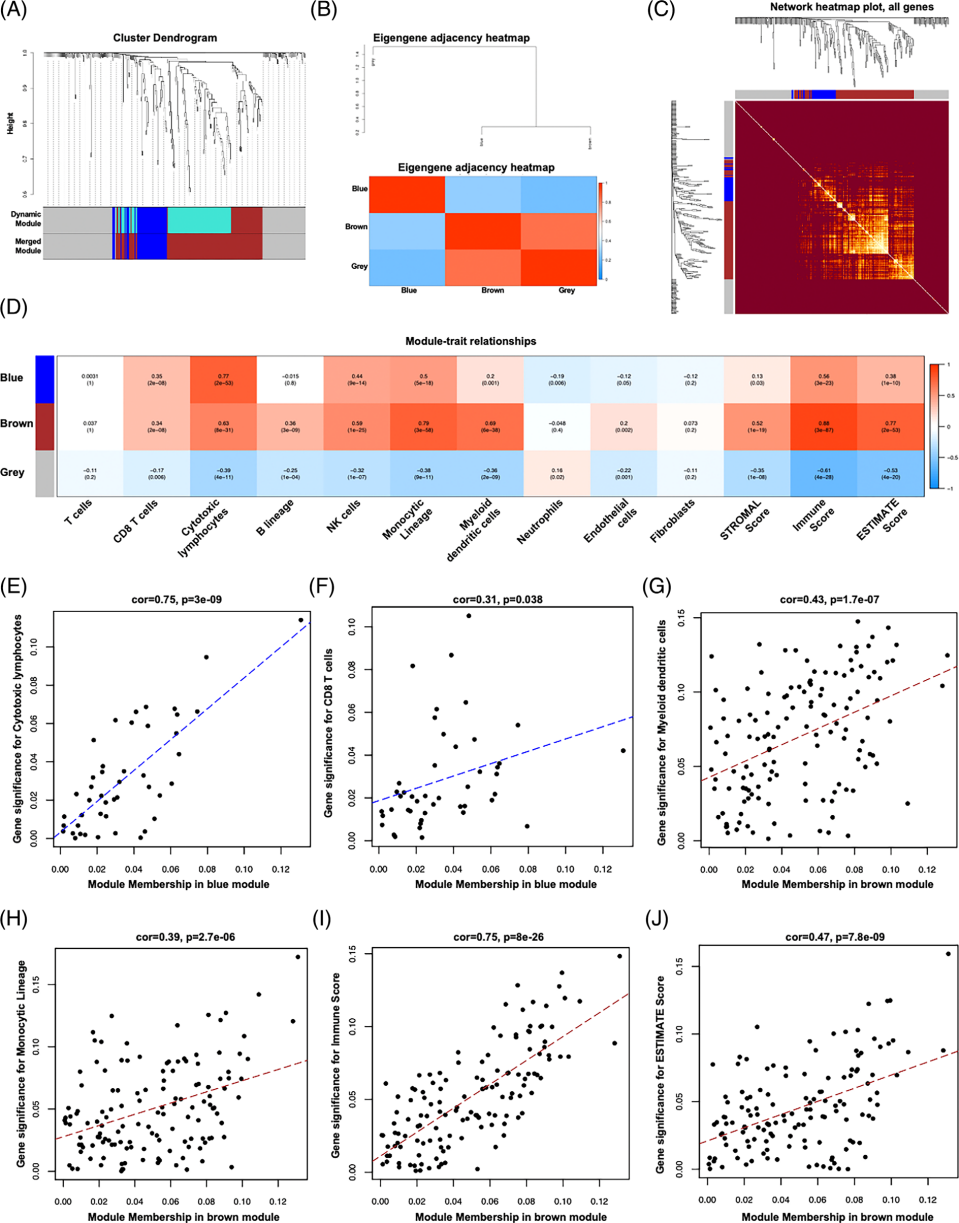
Weighted gene co‐expression network analysis of EBV associated differentially expressed genes (DEGs). Hierarchical cluster tree of the EBV associated DEGs. The assigned modules and genes were represented by the colour bands and the tips of the branches, respectively (A). The Eigengene adjacency heatmap was applied to show the correlation between different modules (B). Heat map shows the values of TOM among the genes of the network delimited in modules with the dynamic method. Low TOM is indicated by brown colour, whereas higher TOM is indicated by progressively luminous yellow colour (C). Correlation between module genes and TIICs and ESTIMATE score. Module characteristic genes and immune characteristic scores were evaluated by Pearson correlation coefficient, and the number in brackets indicates the *p*‐value (D). Relationship between gene significance (GS) and different module memberships (MMs). Scatter plot showed the association between GS and MM. The blue module showed significant GS‐MM with cytotoxic lymphocytes (E) and CD8 T cells (F). The brown module has positive association with GS in monocyticlineage (G), myeloid dendritic cells (H), IMMUNE score (I) and ESTIMATE score (J).

To identify key gene modules relating to immune infiltration, all DEGs between EBV+ versus EBV‐ GC tissues were employed for building hierarchical clustering trees. Only one outlier sample was found (Figure S3A). Next, we performed co‐expression analysis to construct the network of co‐expression. To ensure a scale‐free network, the power of β = 8 was set as the soft thresholding power (Figure S3B,C). Three co‐expression modules were identified, which were displayed by blue, brown and grey (Figure 3A). The correlated heatmap was shown in Figure 3B. Moreover, the TOM value of the heat map was shown among the gene expression of the network delimited in modules by the dynamic method (Figure 3C). The grey module showed low TOM, and the brown module showed higher TOM. To further evaluate the association between modules and immune infiltration, different immune cells (MCP algorithm) and ESTIMATE scores were used in cluster analysis. As shown in Figure 3D, the brown module was positively associated with CD8 T cells, cytotoxic lymphocytes, B lineage, NK cells, monocyticlineage, myeloid DCs, endothelial cells and all ESTIMATE scores. Blue module was positively associated with CD8 T cells, cytotoxic lymphocytes, NK cells, monocyticlineage, myeloid DCs, and all ESTIMATE scores. However, grey modules, which were unassigned genes, mostly showed a negative correlation with immune cells and ESTIMATE scores. All module genes were presented in Table S6.

### 3.4 Relationship between GS and different module memberships

Because the blue module has a close module‐trait relationship with cytotoxic lymphocytes and CD8 T cells, and the brown module was more obvious in monocyticlineage, myeloid DCs, IMMUNE score and ESTIMATE score. We investigated the GS and MM between them. As shown in Figures 3E,F, the blue module showed significant GS‐MM with cytotoxic lymphocytes (Cor = 0.75, *p* = 3 E‐09) and CD8 T cells (Cor = 0.31, *p* = 0.038). In addition, the brown module has a positive association with GS in monocyticlineage (Cor = 0.43, *p* = 1.7 E‐07, Figure 3G), myeloid DCs (Cor = 0.39, *p* = 2.7 E‐06, Figure 3H), immune score (Cor = 0.75, *p* = 8 E‐26, Figure 3I) and ESTIMATE score (Cor = 0.47, *p* = 7.8 E‐09, Figure 3J).

### 3.5 Immune‐associated genes in EBVaGC

**FIGURE 4:**
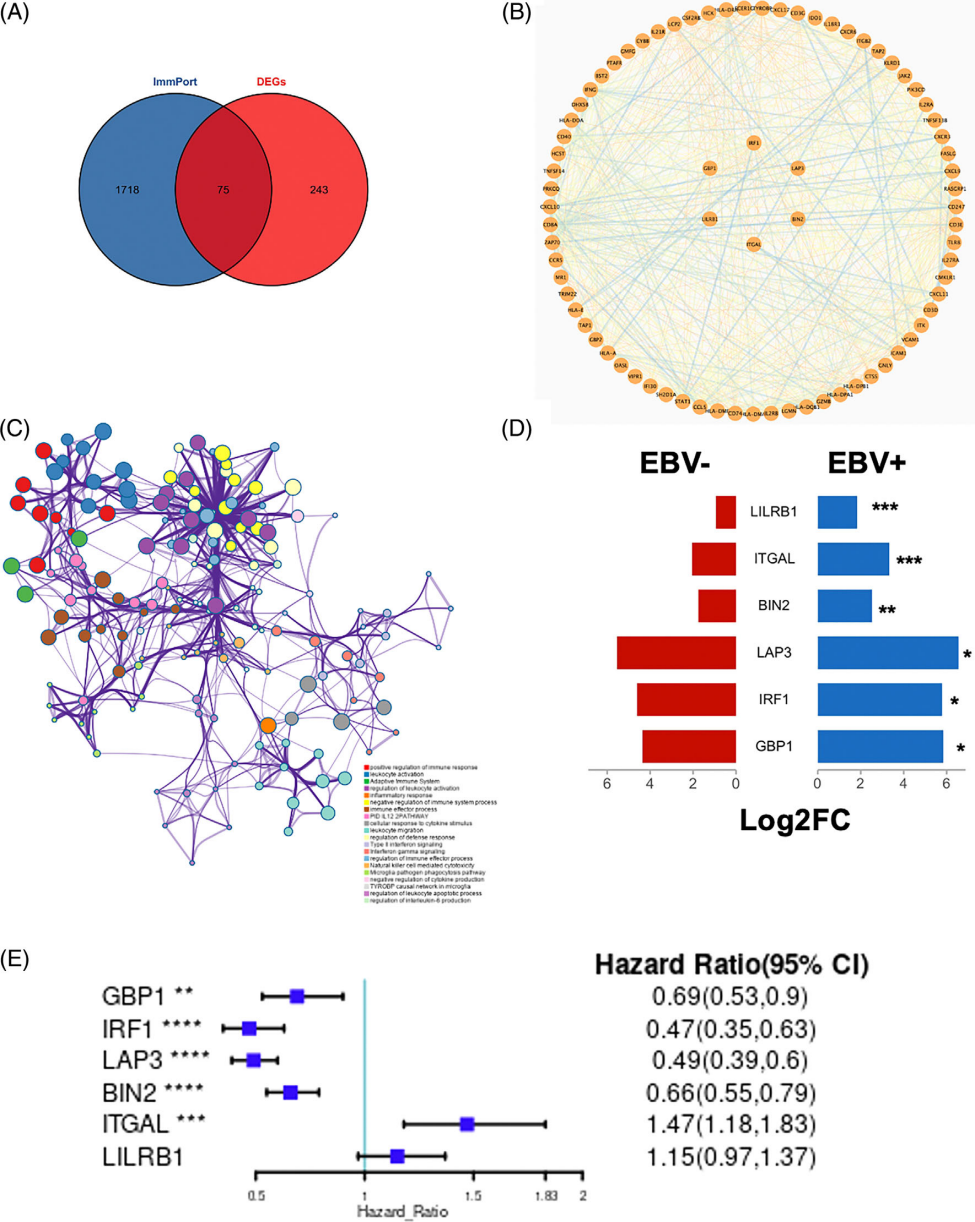
Functional analysis of hub genes. Venn diagram showed the common genes within ImmPort and DEGs (A). The relationship between these genes was presented by the PPI network, which was performed by cytoscape 3.7.2 (B). A subset of GO analysis was carried out to analyse the functional enrichment (C). To explore the association between key hub genes and gastric cancer, the mRNA expression of six key hub genes between EBV+ and EBV‐ GC tissues were given (D). The forest plots showed the prognosis analysis of the six key hub genes in patients with GC (E). *p < 0.05; **p < 0.01; ***p < 0.001; ****p < 0.0001.

To identify the key immune‐related genes in EBVaGC, the DEGs were fetched intersected genes with the immune‐related gene list from the Immport. As shown in Figure 4A, a total of 75 genes were identified as key immune‐related genes in EBVaGC. The PPI network showed the relationship between these genes, the six genes of the inner ring, including interferon regulatory factor 1 (IRF1), leucine aminopeptidase 3 (LAP3), bridging integrator 2 (BIN2), integrin subunit alpha L (ITGAL), leucocyte immunoglobulin‐like receptor B1 (LILRB1) and guanylate binding protein 1 (GBP1), were the top three hub genes of blue and brown modules (Figure 4B).

### 3.6 Hub genes identification

To identify the critical components within these modules, genes with |*k*_ME_| ≥ 0.7 were considered as hubs in each respective module. As shown in Table S7, all |*k*_ME_| ≥ 0.7 hub genes were presented. The blue and brown modules included 29 and 89 hub genes, respectively. A subset of GO‐enriched terms has been selected and rendered as a network plot. These hub genes were enriched in positive regulation of immune response, leucocyte activation, adaptive immune system, regulation of leucocyte activation and inflammatory response, et al. (Figures 4C and S4A). Then, the PPI enrichment analysis has been carried out for analysis of physical interactions. The MCODE algorithm has been applied to identify densely connected network components. As shown in Figure S4B, these hub genes were divided into four MCODE parts. Pathway and process enrichment analysis has been applied to each MCODE component independently, and the three best‐scoring terms by *p*‐value have been retained as the functional description of the corresponding components, shown in the tables underneath corresponding network plots within Figure S4C and Table S8. To analysis which signalling pathways were involved in the regulation of these hun genes, the KEGG analysis was performed. As shown in Figure S5, the top five associated signalling pathways were NFKAPPAB 01, PEA3 Q6, CREL 01, NFKB C and ELF1 Q6. The top three hub genes of blue (GBP1 [guanylate binding protein 1], IRF1 [interferon regulatory factor 1], LAP3 [leucine aminopeptidase 3]) and brown (BIN2 [bridging integrator 2], ITGAL [integrin subunit alpha L], LILRB1 [leucocyte immunoglobulin‐like receptor B1]) modules were selected as representative genes into next analysis.

### 3.7 The association between key hub genes and GC

As exemplified in Figure 4D, all key hub genes were overexpressed in EBVaGC samples. Interestingly, as presented in Figure 4E, high expressed GBP1 (HR = 0.69, 95% CI = 0.53–0.9), IRF1 (HR = 0.47, 95% CI = 0.35–0.63), LAP3 (HR = 0.49, 95% CI = 0.39–0.6) and BIN2 (HR = 0.66, 95% CI = 0.55–0.79) suggested benign prognosis of patients with GC. However, high expression of ITGAL was association with a poor prognosis (HR = 1.47, 95% CI = 1.18–1.83).

**FIGURE 5:**
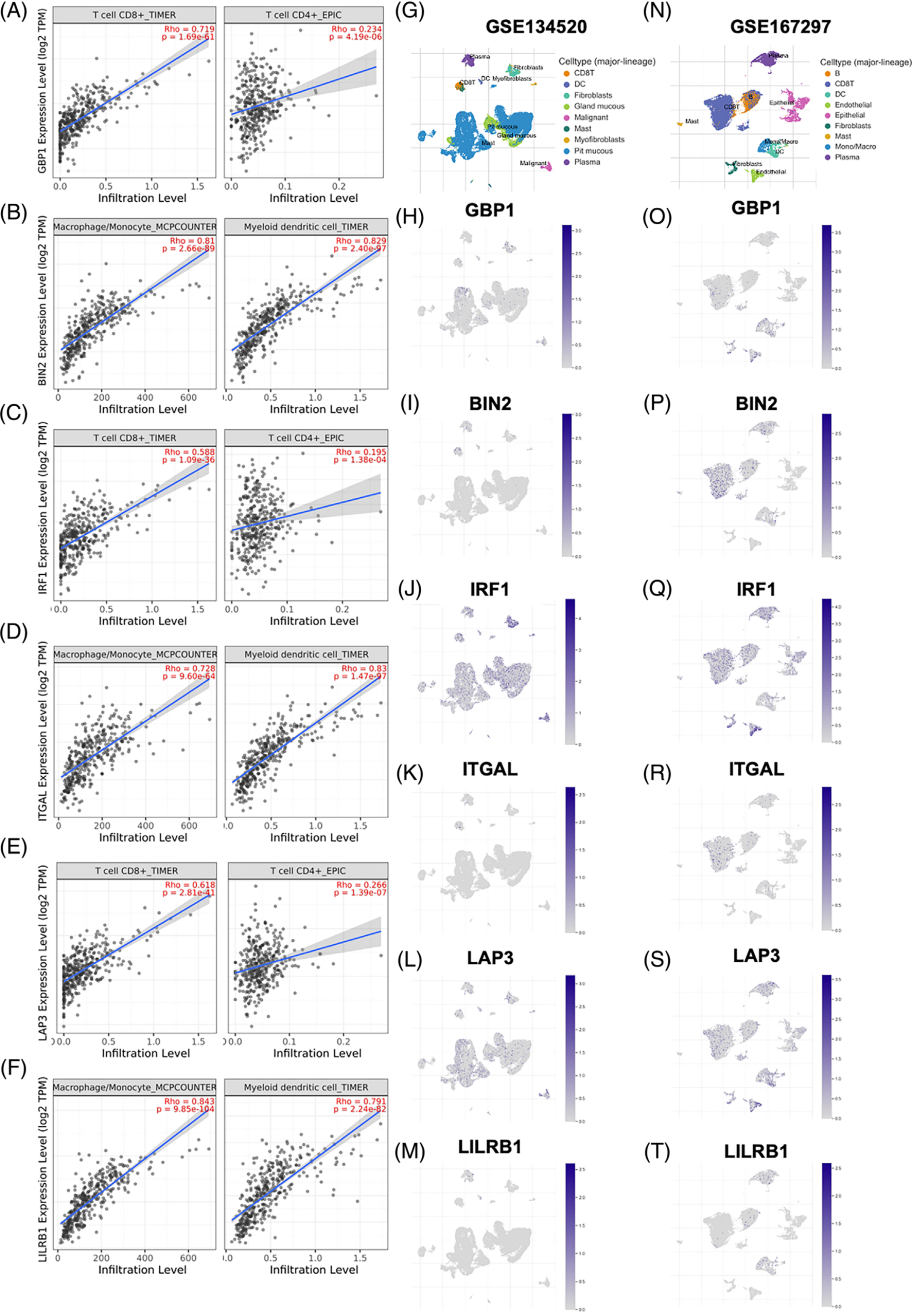
The association between distribution and expression of key hub genes and cell subsets in gastric cancer. The scatter plots showed the association between CD8+/CD4+ T cells and GBP1 (A), IRF1 (C) and LAP3 (E). The scatter plots showed the association between macrophage–monocyte/myeloid dendritic cells and BIN2 (B), ITGAL (D) and LILRB1(F). The cell subset profiles (UMAP) of GSE134520 (G) and GSE167297 (N) were given. The expression of GBP1 (H and O), BIN2 (I and P), IRF1 (J and Q), ITGAL (K and R), LAP3 (L and S) and LILRB1(M and T) in cell subsets were presented.

To further confirm these key hub genes were associated with profiles of TIICs, the TIMER database was applied. GBP1 (Figure 5A), IRF1 (Figure 5B) and LAP3 (Figure 5C), which from blue modules were positively associated with CD8+ T cells and CD4+ T cells. In addition, high expression of the brown module genes, including BIN2 (Figure 5D), ITGAL (Figure 5E) and LILRB1 (Figure 5F), was associated with increased ratios of macrophages/monocytes and myeloid DCs. To further explore the expression of key hub genes in different cell types in GC, a single‐cell sequencing method with two datasets was adopted. The landscapes of GC cells in GSE134520 (Figure 5G) and GSE167297 (Figure 5N) were given. GBP1‐positive cells were distributed in pit mucous, fibroblasts, mono/macro and plasma (Figure 5H and O). BIN2 and ITGAL were mainly expressed in CD8+ T cells (Figure 5I,K,P,R). IRF1 was highly expressed in various immune cells and tumour cells (Figure 5J,Q **)**. LAP3 was widely expressed in DCs, endothelial cells, fibroblasts and malignant cells (Figure 5L,S **)**. LILRB1‐positive cells were mainly associated with DC (Figure 5M,T **)**.

**FIGURE 6:**
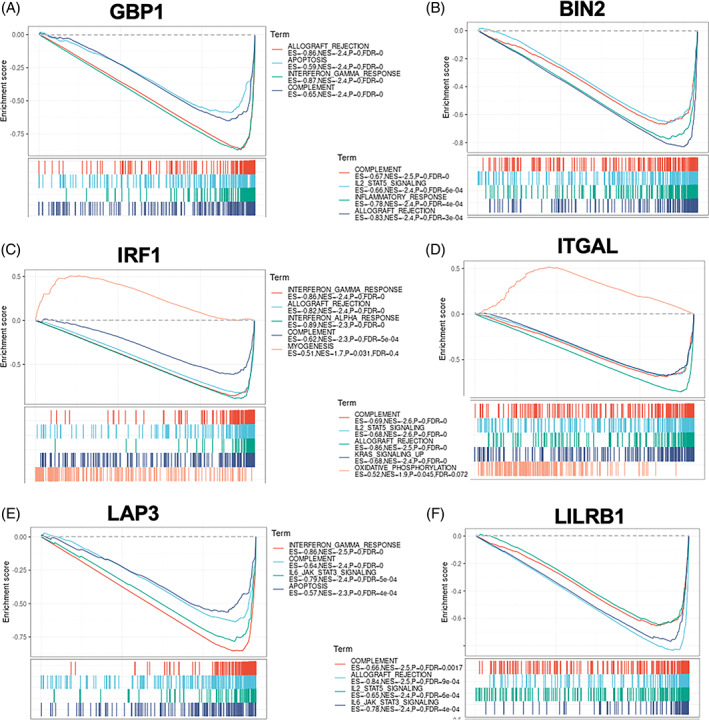
Immune‐related pathways could be activated by each of the six key hub genes in gastric cancer. The TCGA‐STAD expression dataset was used, and the cutoff values were set at 35%. The gene set enrichment analysis of GBP1 (A), BIN2 (B), IRF1 (C), ITGAL (D), LAP3 (E) and LILRB1(F)

Then, we investigated the potential functions of these key hub genes by using GSEA. Interestingly, high expression of each blue module genes was all positively associated with interferon‐gamma response (Figure 6A,C,E). In addition, higher levels of brown module genes were associated with the activation of the complement system and IL‐2‐STAT5 signalling pathway (Figure 6B,D,F). These results suggested that these representative module genes are associated with the immunoregulation of GC.

### 3.8 GBP1, BIN2 and LAP3 are overexpressed in EBVaGC tumour tissues

**FIGURE 7:**
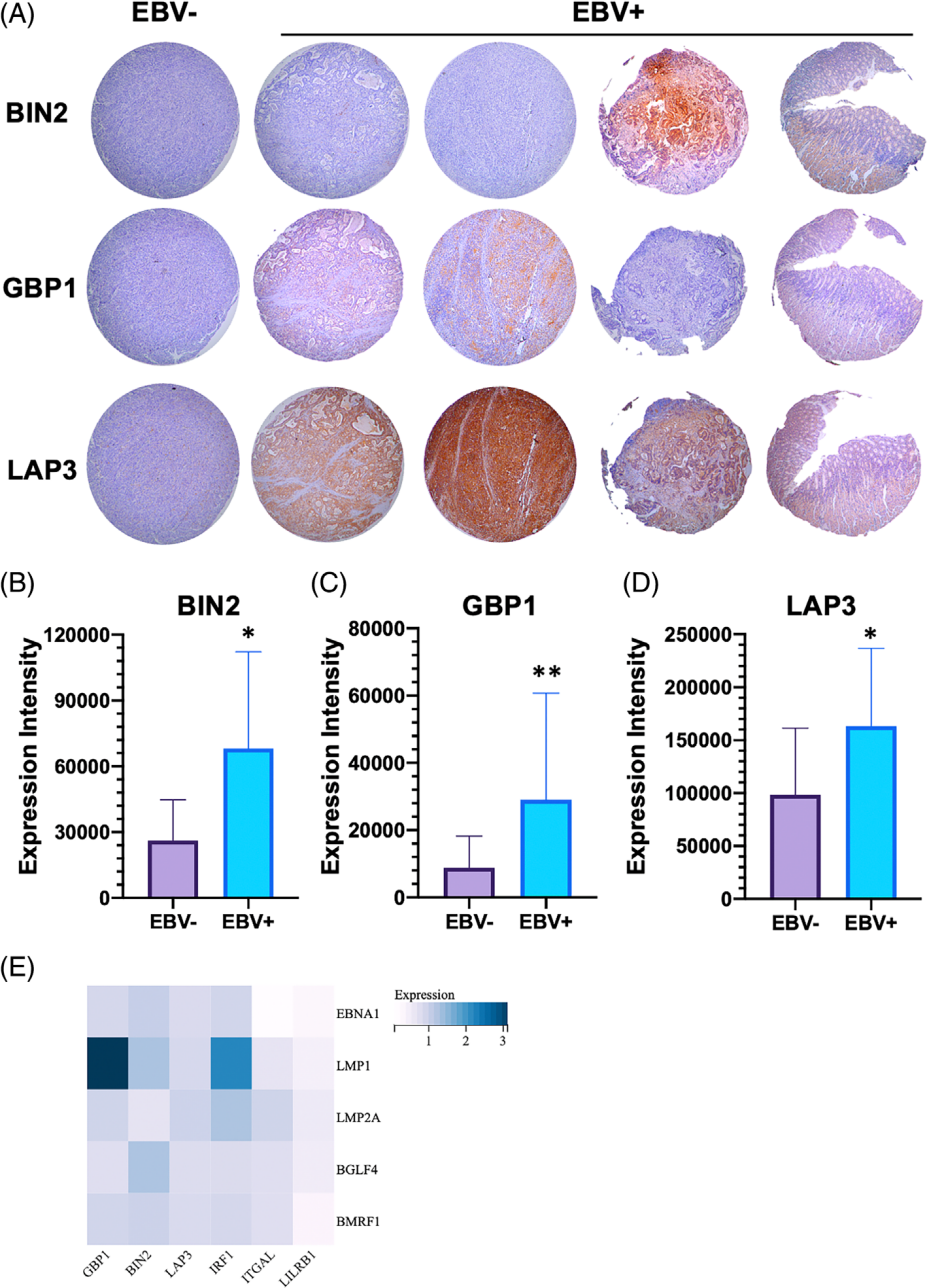
Expression of key hub genes and regulation of latent/lytic proteins of EBV expression in gastric tissue. Immunohistochemistry assays were performed to detect expression and distribution of GBP1, BIN2 and LAP3 proteins in gastric tissue microarray (A). Quantitative analysis results are presented (B–D). The pSG5‐EBNA1, pSG5‐LMP1, pSG5‐BMRF1, pSG5‐BGLF4 and pcDNA3.1‐LMP2A were transfected into MGC‐803 cells. Then, the mRNA levels were determined by the real‐time PCR method. The expression of associated gene was normalized to GAPDH by using a △△GA method (E). *p < 0.05; **p < 0.01.

To detect the expression profiles of key hub genes in GC tissues, we investigate the expression and distribution of GBP1, BIN2 and LAP3 by using IHC in GC tissue microarray. Totally, 204 GC tissues were included and investigated by Guilin Medical University. Among these 201 GC tissues, 9.45% of samples are EBV‐positive (19 cases). As shown in Figure 7A, both GBP1 and BIN2 and LAP3 were mainly expressed in the cytoplasm. The histochemical score was performed on the results of tissue microarray IHC, and the statistical results showed that GBP1, BIN2, and LAP3 were expressed at higher levels in EBV‐positive tissues than in EBV‐negative tissues (Figure 7B,C,D).

### 3.9 LMP1 overexpression modulates the expression of key immune genes in GC

We then explored the effect of multiple EBV viral genes on the expression of these immune genes. As demonstrated in Figure 7E, we observed that compared with control cells, LMP1‐overexpressed MGC‐803 cells had higher levels of GBP1 and IRF1, while the expression of the other four genes was not affected significantly. In addition, EBNA1 overexpression remarkably reduced the expression of ITGAL and LILRB1. It should be noted that several other viral genes, including LMP2A, BGLF4 and BMRF1, had little effect on these immune regulatory molecules.

## 4 DISCUSSION

Interactions between tumour cells and TIICs in the TME are complex and variable among individuals of the same cancer type. The TME is an essential component in the pathogenesis of EBV‐associated malignancies, including EBVaGC. The TIICs compose of multiple cell types, play essential roles in tumour development and immune escape. In this study, we demonstrated that EBV infection alters the profile of immune infiltration in GC. Specifically, EBV infection modulates the proportion of TIICs, as evidenced by the increased proportion of CD8+ T cells, Tregs, macrophage M1, and the decreased proportion of B cell memory, macrophage M0, and DCs resting in EBVaGC tissues. Through WCGNA analysis, the DEGs between EBV‐ and EBV+ GC tissues were divided into two modules. The blue module showed significant GS‐MM with cytotoxic lymphocytes and CD8 T cells. In addition, the brown module has a positive association with GS in monocyticlineage, myeloid DCs, IMMUNE score and ESTIMATE score. In addition, we identified the top three hub genes of blue (GBP1, IRF1 and LAP3) and brown (BIN2, ITGAL and LILRB1) modules as representative genes. These genes are all associated with the distribution of TME in GC. Results of tissue microarray IHC further certificated GBP1, BIN2 and LAP3 were overexpressed in EBVaGC tumour tissues. Importantly, LMP1 expression is account for the overexpression of GBP1 and IRF1.

Although only approximately 10% of gastric carcinoma tissues are EBV‐positive, the distinctive pathological manifestation in EBVaGC promotes researchers to further explore the role and pathogenic mechanism of EBV in GC. A study indicated a large overlap of TIICs interactions between EBV‐ and EBV+ GC tissues, and it is hard to suggest if certain characteristics are EBV‐specific.^20^ Interestingly, through analysing the transcriptome of TCGA‐SWAD datasets and TIICs distribution, we indicated EBV infection has more effects on the immune cells of GC rather than stromal cells. These findings were consistent with the DNA methylome and transcriptome analysis of a previous study.^29^ Our results suggested that the proportion of CD8+ T cells and CD4+ T cells was higher in EBVaGC tissues. This is because the lytic and latent proteins of EBV are rich in antigens that promote the activation of mobilization of specific CD4+ and CD8+ T cells as well as NK cells, leading to antiviral immunity.^18^ In EBV+ GC, only scattered B cells are observed in the stroma.

To further study the mRNA expressed profile changed by EBV infection in GC, three datasets were analysed to screen the DEGs. Unsurprisingly, the up‐regulated genes were clustered in immune and inflammation responses. In an immunocompetent host, EBV infection triggers both innate and adaptive immune responses that subdue the virus and bring the infection under control. In our study, we divided these DEGs into two parts according to the WGCNA algorithm. Interestingly, these two cluster genes are related to different TIICs, respectively. The blue module is associated with cytotoxic lymphocytes and CD8+ T cells, which is reminiscent of recent findings that the CD8+ T cells are infiltrated in greater numbers than CD4+ T cells in EBVaGC.^30^ That is probably because EBV infection significantly activates the CTL signature,^31^ and EBV+ GC cells express more CCL22, which attracts more Treg cells.^32^ The brown module is more closely associated with monocyticlineage and myeloid DCs. Compared with EBV‐negative GC, larger amounts of DCs are present in EBV+ GC tissues.^33^ Mature DCs are close to tumour cells and positively correlated with the abundance of lymphocyte infiltration.^34^

In our study, we have artificially set the top key module genes including GBP1, IRF1, LAP3, BIN2, ITGAL, and LILRB1 as representative EBV‐associated genes. In fact, the associations between EBV and these genes are rarely studied before. In some types of haematological malignancies, IRF4 is an account for the activation of GBP1 in EBV+ tissues instead of EBV‐ tissues.^35^ The real‐time PCR and Western blot results are identified in peripheral blood mononuclear cells in patients with chronic active Epstein–Barr virus infection and control subjects.^36^ IFNγ is a cytokine secreted by tumour–infiltrating T cells and induces PD‐L1 expression by stimulating the JAK/STAT signalling pathway.^37^ In EBVaGC, IFNγ activates JAK2/STAT1/IRF1 signalling pathways and promotes PD‐L1 expression.^38^ Interestingly, this phenomenon depends on the presence of EBNA1.^38^ Our results do not observe the overexpression of EBNA1 can enhance the expression of IRF1 in MGC cells. This may be due to a lack of activation of IFNγ in GC cells. The LAP3 is overexpressed in breast cancer^39^ and hepatocellular carcinoma.^40^ Although we found LAP3 was overexpressed in EBVaGC, and the overexpression of LAP3 was associated with benign prognosis of patients with GC, as well as BIN2. The association between LAP3 or BIN2 and GC was less reported. Bioinformatic analysis indicated that ITGAL expression is strongly associated with numbers of immunological markers including PD1 in GC. Importantly, overexpression of ITGAL indicated a poor prognosis for GC patients.^41^ According to immune infiltration analysis, the high‐expression level of ITGAL was positively correlated with infiltrating degree of CD8+ T cell, B cell, monocytes, neutrophil and macrophage, T‐cell regulatory, NK cells and myeloid DCs.^41^ These findings were similar with our results. In addition, we observed that in GC cells, EBV promotes ITGAL expression and regulates associated immune infiltration. Zhang et al. prove that LILRB1 is associated with M2 macrophage infiltration. In addition, GC patients with high expression of LILRB1 have a poorer prognosis.^42^ However, our results showed a poor tendency of prognosis with LILRB1 expression, and the *p*‐value was still >0.05. Additionally, the expression of LILRB1 was negatively regulated by EBNA1, a key latency protein of EBV.

**FIGURE 8:**
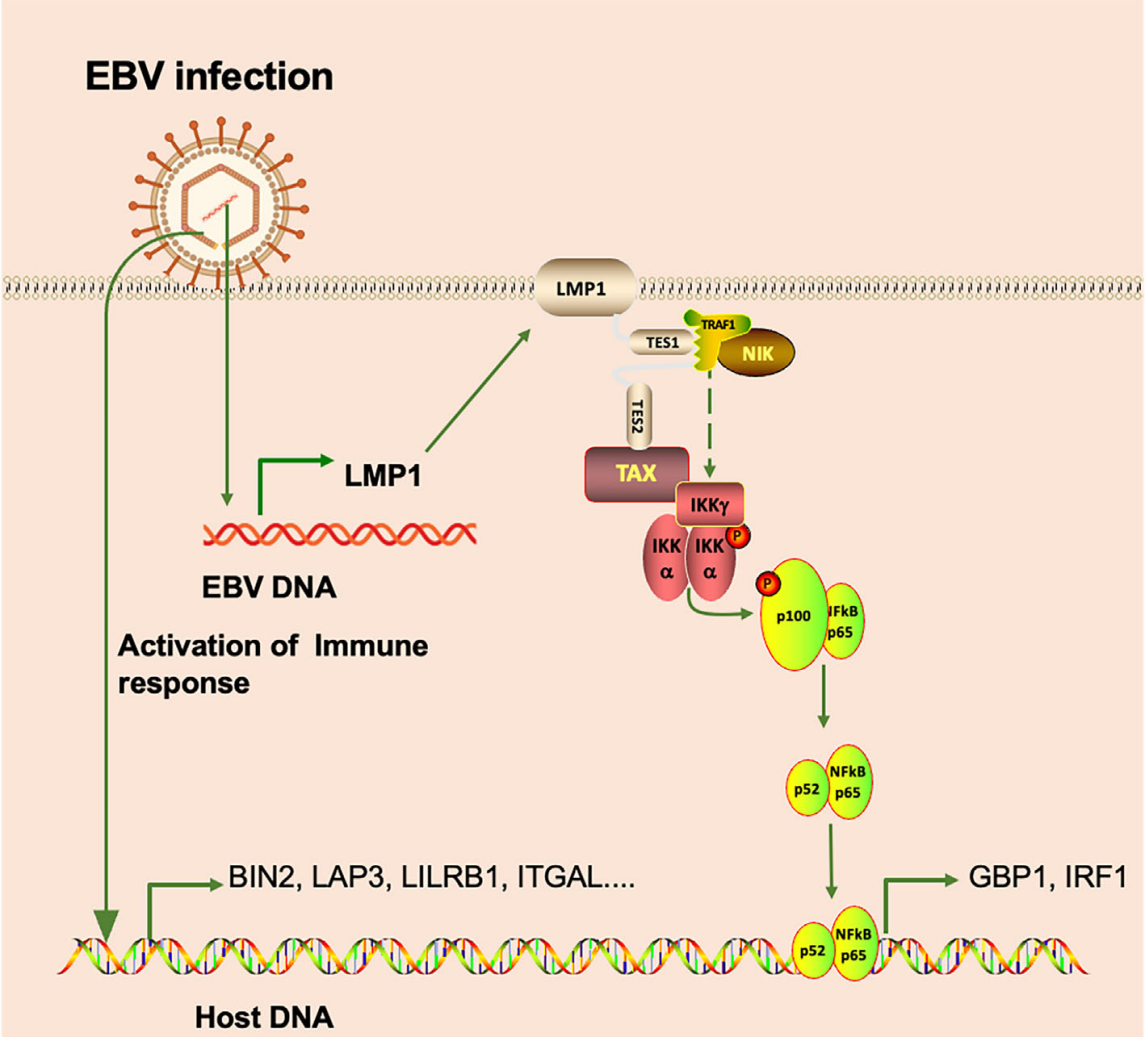
The schematic presentation of EBV infection regulates tumour microenvironment of gastric cancer. The lytic EBV virion infects host cells, activates the immune response of host and promotes expression of associated key hub genes. Then EBV establishes latent infection in host cells and expresses LMP1, which promotes activation of the NF‐κB signalling pathway and expression of GBP1 and IRF1.

Regarding EBVaGC, multiple systematic studies have summarized EBV gene‐expression patterns in gastric carcinomas. Overall, EBVaGC seems to display a unique transcription/latency pattern that does not fit the ‘standard’ EBV latency patterns. A systematic review has demonstrated that the most frequently expressed latent proteins are EBNA1 and LMP2A, followed by lytic proteins, such as BARF0 and BARF1, and the latent proteins LMP1 and LMP2B had lower expressions.^43^ As a representative, EBV promoted GBP1, IRF1, LAP3, BIN2, ITGAL, and LILRB1 overexpression in GC tissues. Then, we explored various EBV latent/lytic key proteins, including EBNA1, LMP1, LMP2A, BGLF4, and BMRF1, to determine their effect on the selected six immune hub genes. Compared with the pSG5 vector control group, LMP1 overexpression significantly enhances the expression of IRF1 and GBP1, while the other four viral proteins have little effect on the expression of IRF1 and GBP1 (Figure 8).

The most important pathways involved in tumour development in EBV‐associated malignancies are the NF‐κB, and the JAK/STAT pathways.^20^ In EBVaGC, the EBV activates CD40 signalling and promotes survival and proliferation.^44^ Interestingly, LMP1 mimics the function of CD40 and interacts with TRAFs to activate NF‐κB.^45^ Previous studies have indicated that LMP1 could activate the NF‐κB signalling pathway, leading to the enhancement of GBP1^46^ and IRF1.^47^, ^48^ In the present study, the common DEGs between EBV‐ and EBV+ GC tissues were mainly associated with the NF‐κB signalling pathway. LMP1 can promote the expression of CCL5 and CCL2 in NPC cells, both of which can recruit tumour‐associated macrophages.^49^ Interestingly, our results indicated that the number of M0 macrophages is higher in EBV‐ GC tissues than that in EBV+ GC tissues, which is consistent with the previous study.^50^ However, EBV may promote M1 polarization of macrophages in EBVaGC.

We noticed that only two genes (GBP1 and IRF1) are regulated by LMP1, and we supposed that there are two main reasons to explain the phenomenon. Firstly, we only selected several key genes from databases, there may be other key genes of EBV that are not activated. Secondly, more than 60% of EBVaGC can be detected with gene expression of EBV lytic infection, such as BARF1, BARTs and BcLF1,^43^ even 100% detection of BARF0.^32^, ^43^, ^51^ These results suggested that EBV virions continuously invade GC tissue and regulate the TME, rather than one or some key viral genes playing a role in EBVaGC.

Several limitations existed in the present study. First, the number of EBV+ GC patients was too few. Although we recruited three datasets to obtain common DEGs, it is hard to avoid sample deviation in the analysis. Second, as analysed above, we only observed that two key module genes are regulated by EBV. Other molecular regulated mechanisms are not found in this study. Therefore, we call on more explorations of molecular mechanisms and clinical research to illustrate the association between EBV infection and TME alteration in GC.

In conclusion, our study indicated that EBV infection changes the TME of GC through primarily regulating the TIICs, and highlights the analytical ability of WCGNA to illustrate the molecular mechanisms by identifying the functional modules and hub genes associated with EBV infection. The top three hub genes of blue (GBP1, IRF1 and LAP3) and brown (BIN2, ITGAL and LILRB1) modules were further analysed as representative genes. Furthermore, EBV‐encoded LMP1 expression is account for the overexpression of GBP1 and IRF1. Importantly, the activation of key immune genes was more dependent on the invasiveness of the whole EBV virion instead of single EBV latent/lytic gene expression.

## AUTHOR CONTRIBUTIONS

Heng Zhou and Shuili Jing performed data collection and wrote this manuscript. Yu Liu performed the cell experiments. Xingxiang Duan and Ruohan Li performed the analysis. Yaoqi Zhu and Hui Wang drawn the pictures. W. S. finished the tables. Xuming Wang and Zhi Zeng performed the IHC assays. Youjian Peng and Yan He performed data curation. Qingsong Ye provided this idea and financial support.

## CONFLICT OF INTEREST

The authors declare that they have no competing interests regarding the publication of this paper.

## Supporting information


**Figure S1.** The MCP‐counter (A) and CIBERSORT algorithm (B) were used to analyse the proportions of tumour‐infiltrating immune cells in EBV‐ and EBV+ gastric cancer tissues. ESTIMATE algorithm was used to calculate the stromal (C) from the TCGA‐SWAD dataset. *p < 0.05; **p < 0.01; ***p < 0.001; ****p < 0.0001.

**Figure S2.** The volcano pictures showed the differentially expressed gene distribution of GSE66229 (A) and TCGA‐SWAD (B). Kyoto Encyclopedia of Genes and Genomes pathway analyses of downregulated differentially expressed genes (C).

**Figure S3.** Sample clustering diagram (A) and determination of the optimal soft threshold. The adjacency matrix is transferred into a topology matrix, and the optimal soft threshold β = 8 is determined in the process of module selection (B) and (C).

**Figure S4.** A subset of the PPI enrichment analysis was carried out to analyse the physical interactions, respectively (A). The Molecular Complex Detection (MCODE) algorithm has been applied to identify densely connected network components (B) and (C).

**Figure S5.** The Kyoto Encyclopedia of Genes and Genomes analysis of all the hub genes.

**Table S1.** The upregulated genes between the GSE551575, GSE66229, and TCGA‐SWAD datasets.
**Table S2**. The downregulated genes between the GSE551575, GSE66229, and TCGA‐SWAD datasets.
**Table S3**. Gene ontology analysis of upregulated differentially expressed genes between EBV+ versus EBV‐ GC tissues.
**Table S4**. Gene ontology analysis of downregulated differentially expressed genes between EBV+ versus EBV‐ GC tissues.
**Table S5**. Kyoto Encyclopedia of Genes and Genomes analysis of DEGs between EBV+ versus EBV‐ GC tissues.
**Table S6**. Weighted gene co‐expression analysis (WGCNA) analysis of all module genes.
**Table S7**. Key hub genes of |*k*_ME_| ≥ 0.7 in each respective module.
**Table S8**: Pathway enrichment analysis of each Molecular Complex Detection (MCODE) component.

## Data Availability

The data that support the findings of this study are openly available in TCGA at https://www.cancer.gov/about-nci/organization/ccg/research/structural-genomics/tcga, reference number [8]; GEO at https://www.ncbi.nlm.nih.gov/geo/, reference number [23, 24]; Kaplan‐Meier plotter at https://www.kmplot.com, reference number [26]; TIMER at https://cistrome.shinyapps.io/timer/, reference number [27].
