# Supplementary figures and images for: Identifying the key genes of Epstein–Barr virus‐regulated tumour immune microenvironment of gastric carcinomas
Source: Cell Prolif. 2022 Dec 14;56(3):e13373. doi: 10.1111/cpr.13373 (PMC9977676; doi:10.1111/cpr.13373)

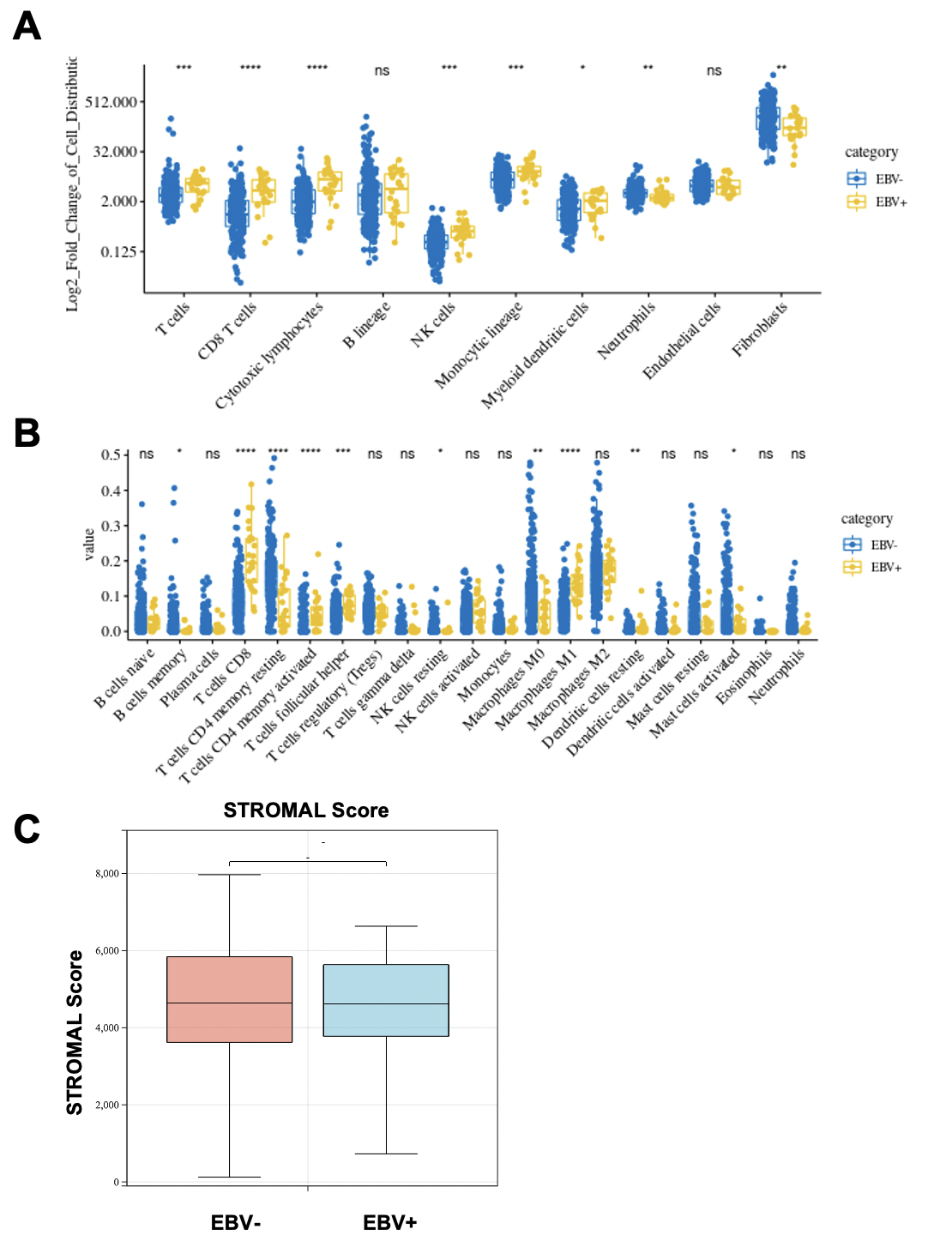

Supplement: Supplementary file 1 — Figure S1. The MCP‐counter (A) and CIBERSORT algorithm (B) were used to analyse the proportions of tumour‐infiltrating immune cells in EBV‐ and EBV+ gastric cancer tissues. ESTIMATE algorithm was used to calculate the stromal (C) from the TCGA‐SWAD dataset. *p < 0.05; **p < 0.01; ***p < 0.001; ****p < 0.0001. [file CPR-56-e13373-s004.png]

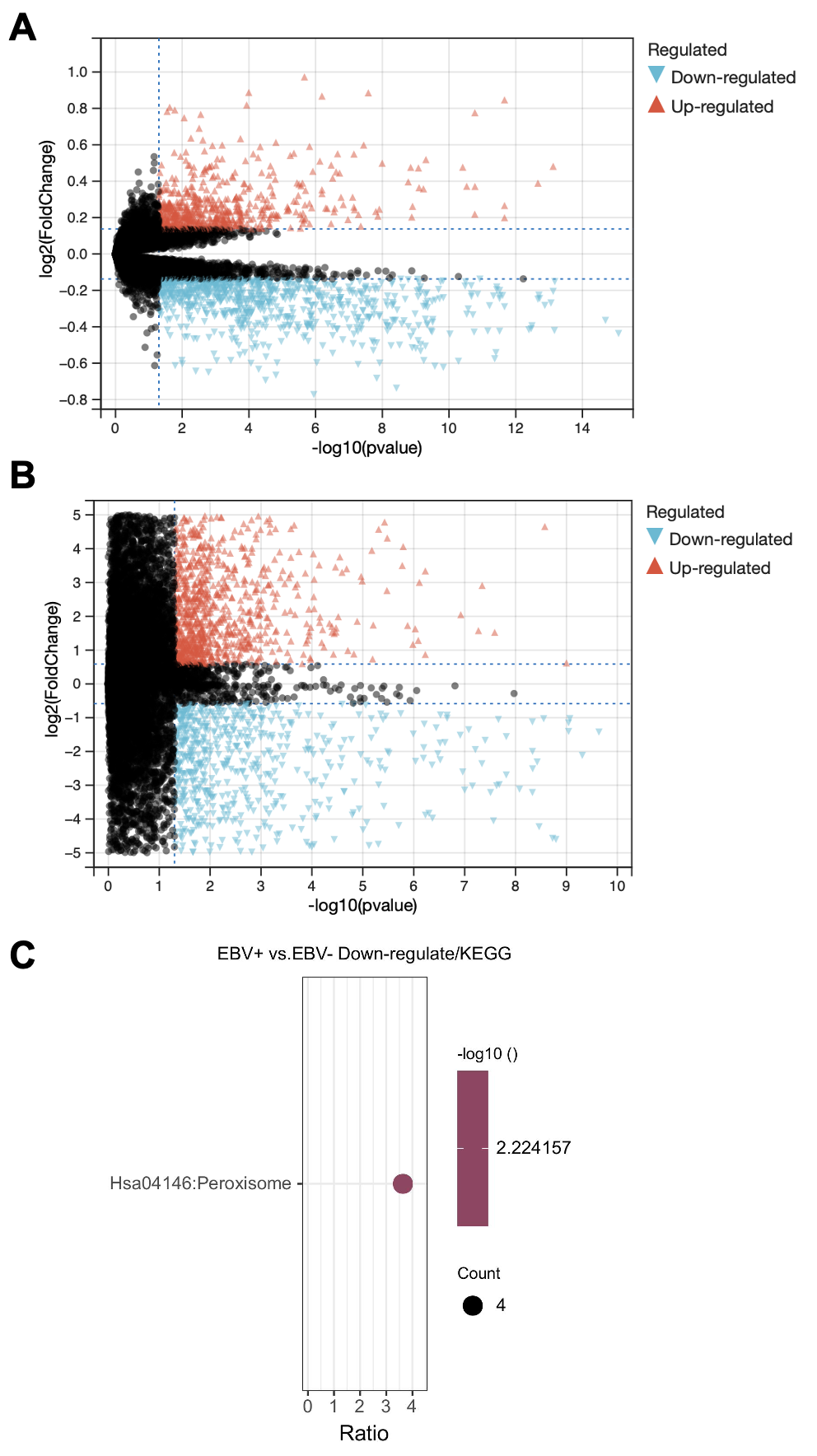

Supplement: Supplementary file 2 — Figure S2. The volcano pictures showed the differentially expressed gene distribution of GSE66229 (A) and TCGA‐SWAD (B). Kyoto Encyclopedia of Genes and Genomes pathway analyses of downregulated differentially expressed genes (C). [file CPR-56-e13373-s001.png]

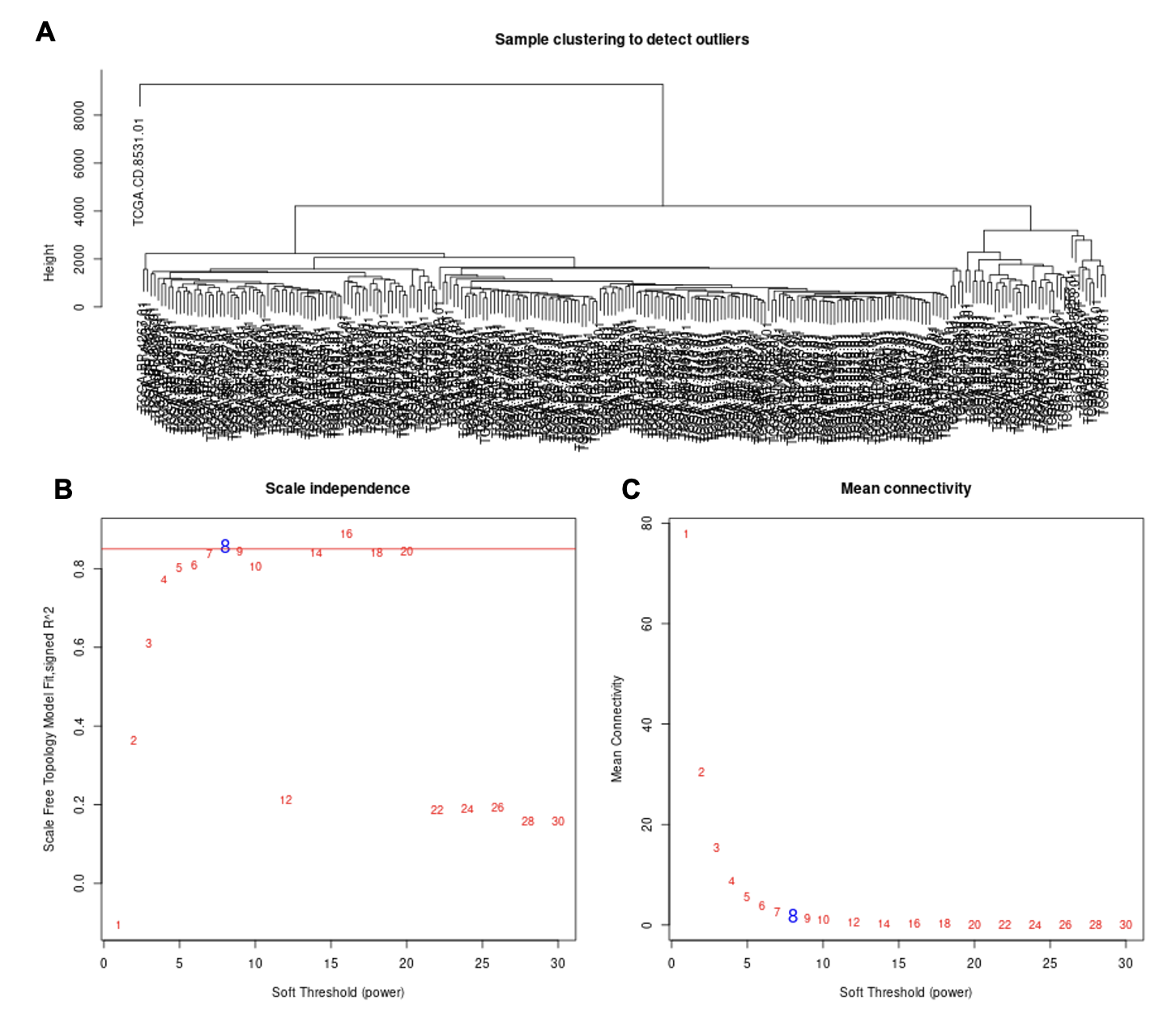

Supplement: Supplementary file 3 — Figure S3. Sample clustering diagram (A) and determination of the optimal soft threshold. The adjacency matrix is transferred into a topology matrix, and the optimal soft threshold β = 8 is determined in the process of module selection (B) and (C). [file CPR-56-e13373-s006.tif]

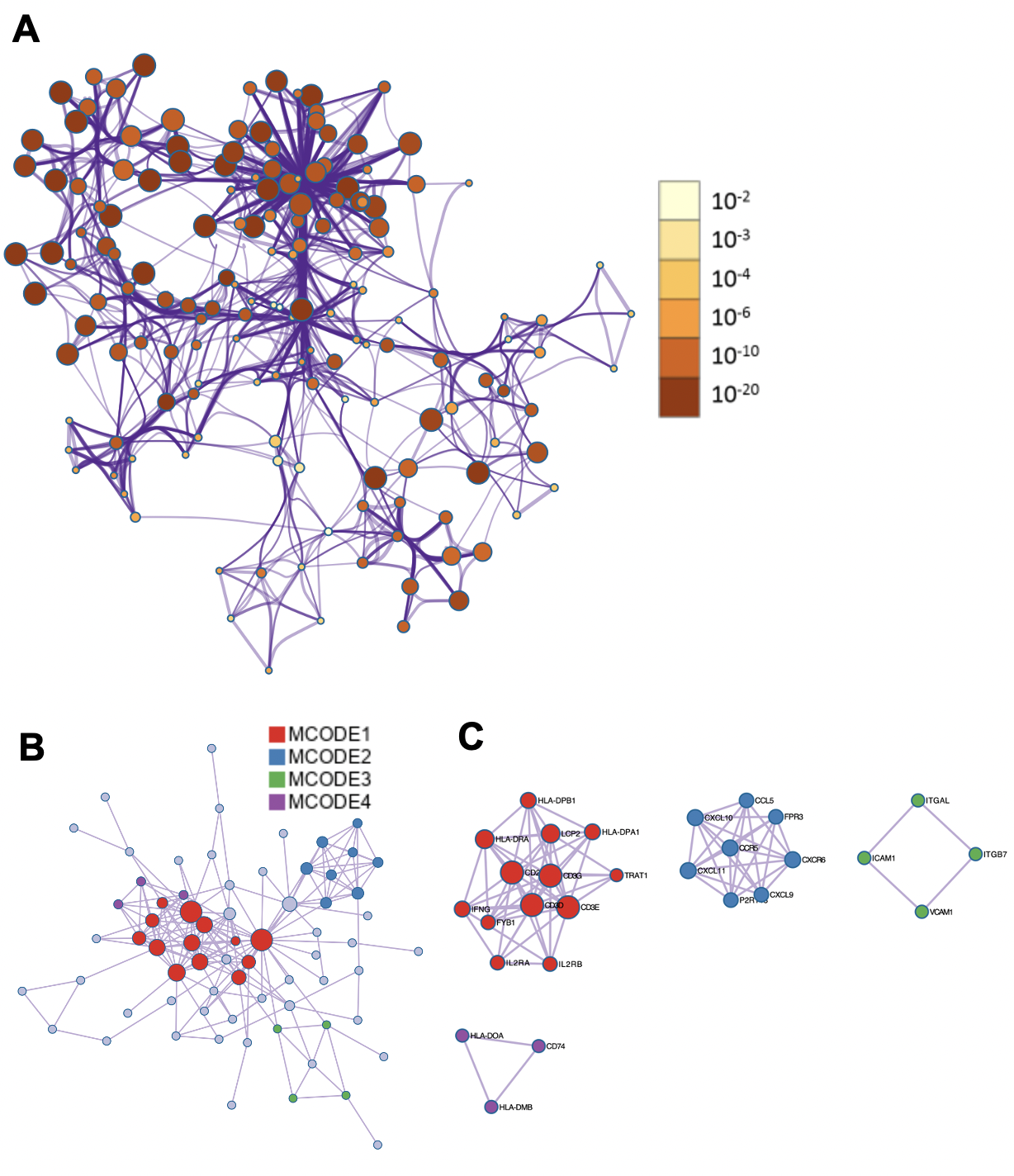

Supplement: Supplementary file 4 — Figure S4. A subset of the PPI enrichment analysis was carried out to analyse the physical interactions, respectively (A). The Molecular Complex Detection (MCODE) algorithm has been applied to identify densely connected network components (B) and (C). [file CPR-56-e13373-s003.png]

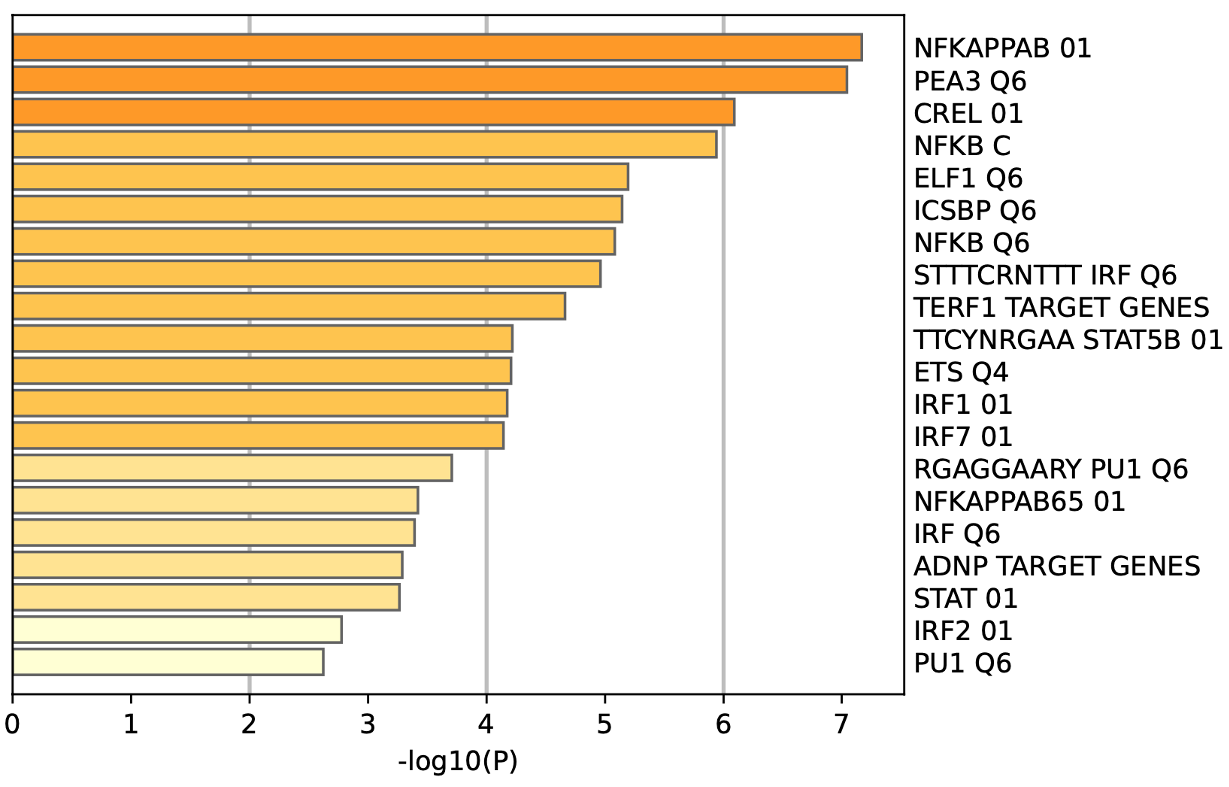

Supplement: Supplementary file 5 — Figure S5. The Kyoto Encyclopedia of Genes and Genomes analysis of all the hub genes. [file CPR-56-e13373-s005.tif]
