# Supplementary material for: Identifying the key genes of Epstein–Barr virus‐regulated tumour immune microenvironment of gastric carcinomas
Source: Cell Prolif. 2022 Dec 14;56(3):e13373. doi: 10.1111/cpr.13373 (PMC9977676; doi:10.1111/cpr.13373)
Supplement: Supplementary file 6 — Table S1. The upregulated genes between the GSE551575, GSE66229, and TCGA‐SWAD datasets. Table S2. The downregulated genes between the GSE551575, GSE66229, and TCGA‐SWAD datasets. Table S3. Gene ontology analysis of upregulated differentially expressed genes between EBV+ versus EBV‐ GC tissues. Table S4. Gene ontology analysis of downregulated differentially expressed genes between EBV+ versus EBV‐ GC tissues. Table S5. Kyoto Encyclopedia of Genes and Genomes analysis of DEGs between EBV+ versus EBV‐ GC tissues. Table S6. Weighted gene co‐expression analysis (WGCNA) analysis of all module genes. Table S7. Key hub genes of |k ME| ≥ 0.7 in each respective module. Table S8: Pathway enrichment analysis of each Molecular Complex Detection (MCODE) component. [file CPR-56-e13373-s002.docx]

**Supplemental Table 1. The upregulated genes between GSE551575, GSE66229 and TCGA-SWAD datasets.**

| Upregulated Genes. |
| --- |
| CD74 HLA-DRA WARS C1QB HLA-DPA1 CLDN18 CXCL17 HLA-A CXCL9 C1QC IFI30 RARRES3 CXCL10 C1QA HLA-E APOC1 BST2 IDO1 CCL5 HLA-DQB1 TAP1 TYROBP FCER1G STAT1 LAP3 UCP2 LGALS9 HLA-DMA UBE2L6 CXCL11 HLA-DPB1 COTL1 ICAM1 LOC400043 GBP4 GBP1 LGMN NKG7 SAMHD1 GZMB CTSS IRF1 GPRC5B PSMB9 LIPA ACP5 GBP5 SLAMF7 EPSTI1 ITGB2 TRIM22 HCST GZMA BIRC3 ALDH3A1 SLC26A9 HLA-DMB PIM2 GM2A HLA-DOA JAK2 OASL CD3D HABP2 OAS2 CD38 ADAM28 HLA-DQB2 CD3E GZMH GNLY PSMB10 FAM26F CECR1 CD8A GAA MAN1A1 SOX2 MS4A6A CYBB IL2RB HAPLN3 IL18BP ADAMDEC1 GBP2 CD2 TAP2 CAMK2N2 FYB SLAMF8 AIF1 GIMAP4 VCAM1 PTAFR ZNF683 BATF SLC15A3 ETV7 CD40 LAMP3 SLCO2B1 CXCR6 CSF2RB SLC9A7 APOL3 PLA1A STAT2 FPR3 ITGAL LCP2 TNFSF13B IGSF6 LAG3 LST1 GIMAP7 PLA2G7 GZMK MS4A7 LAIR1 GMFG IL27RA BTN3A2 NCKAP1L BTN3A1 HIPK2 CXCR3 TMEM140 AOAH TBXAS1 SUSD4 HCK CCR5 FUT8 APOL4 DOK2 IL4I1 FAM46C ITGB7 HAVCR2 MYO1F CA13 CHIT1 TBC1D10C GPR171 COL27A1 PREX1 LILRB4 CD96 CD3G COL22A1 DHX58 IL2RA GIMAP2 GIMAP6 BIN2 RASAL3 GZMM ARHGAP9 ACAP1 IFNG CD247 SIDT1 NFAM1 SLC31A2 PTPN22 CD300LF SIGLEC1 CMKLR1 PIK3CD VASH1 CD84 ZAP70 IRAK3 PRKCQ RASGRP1 ITK LAX1 TNFAIP8L2 KLRD1 FASLG LILRB1 FAM78A TLR8 P2RY13 PYHIN1 TRAT1 IL21R RASGEF1B SH2D1A BTN2A2 NLRC3 AMICA1 TIGIT SIGLEC8 MR1 THEMIS GIMAP8 TNFSF14 KCNA3 EMR2 KLHL6 GNGT2 MIAT IL18R1 DOCK10 PSTPIP1 UBASH3A CRTAM |

**Supplemental Table 2. The downregulated genes between GSE551575 GSE66229 and TCGA-SWAD datasets.**

| Downregulated Genes |
| --- |
| HOXA6 RIMKLA BCL2L10 FAM103A1 LYSMD1 C17orf100 SLC16A10 RAB6B PLA2R1 THAP10 DEPDC7 NEURL2 C11orf74 PPP1R9A PRPF40B RIBC2 ABLIM2 LRRC6 MFSD6L GAS2L3 KBTBD7 NAGS ARL13B PXMP4 FAIM CENPQ TRMT12 FAH NT5DC3 AMIGO1 SLC22A18AS EPHX4 RASGEF1A TNNC2 NINL STK32C LRRC8E KCMF1 HYLS1 PHF6 PDE9A MPP6 TFAP2C PTPRG HOXA11 RCOR2 SLC22A3 TMEM117 LRRC58 LZTFL1 FOXD1 MPV17L TMEM65 KBTBD11 PLEKHG3 MLYCD NUDT19 ZNF480 CGREF1 HOXA5 RMND5A HOXB5 VIPR1 HOXB3 TPD52L1 PKN3 OPN3 TMEM52 KHDRBS3 F12 ECHDC2 NOL3 GOLT1A CIB2 SLC25A4 MYO1B NMB SNTA1 ME1 HOXA13 TLCD1 TPBG HOXA10 RAB15 GRIN2D C1orf115 DNAJA4 CERK HOXB7 HOXC10 ESD SCRN1 DPP4 HEBP1 C4orf48 DSC2 PPIC EFNA1 IRS2 GPC1 PODXL2 REEP6 SLC6A8 PFN2 DUSP23 DSG2 LAPTM4B CAMK2N1 ARL6IP1 CLDN3 |

**Supplemental Table 3. Go analysis of upregulated DEGs between EBV+ vs. EBV- GC tissues.**

| Category | Term | Count | PValue | Genes | Fold Enrichment | Bonferroni | Benjamini | FDR |
| --- | --- | --- | --- | --- | --- | --- | --- | --- |
| BP | GO:0006955~immune response | 45 | <0.001 | ADAMDEC1, CXCL9, CD40, IGSF6, LST1, PTAFR, FASLG, SAMHD1, FYB, CTSS, TNFSF13B, IL27RA, HLA-DMA, HLA-DMB, CCL5, GBP2, CCR5, HLA-DOA, TRIM22, CMKLR1, HLA-DPA1, CD74, CD96, TNFSF14, GZMA, HLA-A, MR1, LAX1, GZMH, HLA-E, CHIT1, CXCL10, ZAP70, CXCL11, IFNG, OAS2, CD8A, IL2RA, HLA-DPB1, HLA-DRA, LCP2, IL18R1, HLA-DQB2, C1QC, HLA-DQB1 | 9.496 | <0.001 | <0.001 | <0.001 |
| BP | GO:0060333~interferon-gamma-mediated signaling pathway | 21 | <0.001 | VCAM1, STAT1, PTAFR, HLA-A, IFI30, ICAM1, HLA-E, OASL, HCK, IFNG, OAS2, IRF1, HLA-DPB1, HLA-DRA, JAK2, GBP2, GBP1, TRIM22, HLA-DQB2, HLA-DPA1, HLA-DQB1 | 26.274 | <0.001 | <0.001 | <0.001 |
| BP | GO:0002250~adaptive immune response | 23 | <0.001 | ZNF683, ITK, CD84, BTN3A1, TRAT1, SH2D1A, CRTAM, TAP2, THEMIS, TAP1, PIK3CD, LILRB1, LAX1, LILRB4, CTSS, HLA-E, ZAP70, IFNG, LAMP3, SLAMF7, JAK2, LAIR1, HAVCR2 | 13.802 | <0.001 | <0.001 | <0.001 |
| BP | GO:0050776~regulation of immune response | 24 | <0.001 | CD96, CD40, VCAM1, SH2D1A, ITGB2, CRTAM, LILRB1, CD3G, HLA-A, ITGAL, CD3E, CD3D, ICAM1, HLA-E, TYROBP, CD8A, IRF1, SLAMF7, ITGB7, KLRD1, CD247, CD300LF, HCST, LAIR1 | 11.971 | <0.001 | <0.001 | <0.001 |
| BP | GO:0050852~T cell receptor signaling pathway | 22 | <0.001 | ITK, BTN3A1, TRAT1, THEMIS, PIK3CD, PTPN22, CD3G, CD3E, CD3D, PSMB10, FYB, PSMB9, ZAP70, IFNG, HLA-DPB1, HLA-DRA, LCP2, PRKCQ, CD247, HLA-DQB2, HLA-DPA1, HLA-DQB1 | 13.205 | <0.001 | <0.001 | <0.001 |
| CC | GO:0042613~MHC class II protein complex | 10 | <0.001 | CD74, HLA-DMA, HLA-DMB, HLA-DPB1, HLA-DRA, HLA-A, HLA-DOA, HLA-DQB2, HLA-DPA1, HLA-DQB1 | 41.41 | <0.001 | <0.001 | <0.001 |
| CC | GO:0009897~external side of plasma membrane | 19 | <0.001 | CD74, CXCL9, LAG3, CD40, VCAM1, FCER1G, FASLG, LILRB1, CD3E, ICAM1, CD2, CXCL10, IFNG, CD8A, IL2RA, CXCR3, IL2RB, KLRD1, CCR5 | 8.124 | <0.001 | <0.001 | <0.001 |
| CC | GO:0009986~cell surface | 25 | <0.001 | CD40, ITGB2, ITGAL, ICAM1, HLA-DMA, CD38, ITGB7, CCR5, TIGIT, HAVCR2, HLA-DPA1, CD74, FCER1G, VCAM1, NFAM1, HLA-A, HLA-E, CD2, BST2, GPRC5B, TYROBP, HLA-DPB1, HLA-DRA, SLC26A9, HCST | 4.208 | <0.001 | <0.001 | <0.001 |
| CC | GO:0071556~integral component of lumenal side of endoplasmic reticulum membrane | 8 | <0.001 | CD74, HLA-DPB1, HLA-DRA, HLA-A, HLA-DQB2, HLA-DPA1, HLA-E, HLA-DQB1 | 25.139 | <0.001 | <0.001 | <0.001 |
| CC | GO:0016021~integral component of membrane | 94 | <0.001 | CD40, LST1, MS4A7, GIMAP2, CD3G, FASLG, CD3E, ITGAL, TMEM140, CD3D, TNFSF13B, ICAM1, IL27RA, SLCO2B1, LAMP3, GPR171, ACP5, CD38, MAN1A1, ITGB7, HLA-DOA, CCR5, HAVCR2, HLA-DPA1, CMKLR1, CD96, LAG3, FCER1G, BTN3A1, NFAM1, GAA, TAP2, CYBB, TAP1, HLA-A, BTN3A2, MR1, LAX1, HLA-E, ALDH3A1, MS4A6A, GPRC5B, TYROBP, CD8A, SLC9A7, SIDT1, CLDN18, TLR8, HCST, HLA-DQB2, HLA-DQB1, IGSF6, PTAFR, CSF2RB, FPR3, CXCR6, APOL4, HLA-DMA, FUT8, ADAM28, HLA-DMB, CXCR3, UCP2, SLAMF8, SUSD4, IL21R, SLAMF7, CD300LF, TIGIT, SLC15A3, LAIR1, P2RY13, CD74, VCAM1, BTN2A2, TNFSF14, SLC31A2, RARRES3, NKG7, LILRB1, LILRB4, CD2, BST2, IL2RA, TBXAS1, HLA-DPB1, HLA-DRA, FAM26F, KLRD1, CD247, SLC26A9, SIGLEC1, SIGLEC8, IL18R1 | 1.657 | <0.001 | <0.001 | <0.001 |
| MF | GO:0032395~MHC class II receptor activity | 7 | <0.001 | HLA-DMA, HLA-DPB1, HLA-DRA, HLA-DOA, HLA-DQB2, HLA-DPA1, HLA-DQB1 | 41.906 | <0.001 | <0.001 | <0.001 |
| MF | GO:0042605~peptide antigen binding | 7 | <0.001 | HLA-DPB1, HLA-DRA, TAP1, HLA-A, HLA-DPA1, HLA-E, HLA-DQB1 | 22.441 | 0.001 | <0.001 | <0.001 |
| MF | GO:0004872~receptor activity | 13 | <0.001 | CD40, ITGB2, CRTAM, CSF2RB, LILRB4, ICAM1, CD2, CXCR3, TLR8, ITGB7, TIGIT, IL18R1, CMKLR1 | 5.373 | 0.002 | 0.005 | 0.005 |
| MF | GO:0005102~receptor binding | 16 | <0.001 | ITK, TNFSF14, CRTAM, FASLG, HLA-A, FYB, TNFSF13B, HLA-E, CD2, CXCL10, ZAP70, HCK, TYROBP, TIGIT, JAK2, HCST | 4.06 | 0.003 | 0.007 | 0.007 |
| MF | GO:0023026~MHC class II protein complex binding | 5 | <0.001 | CD74, HLA-DMA, HLA-DMB, HLA-DRA, HLA-DOA | 28.062 | 0.00748096 | 0.0015018 | 0.00145875 |

**Supplemental Table 4. Go analysis of downregulated DEGs between EBV+ vs. EBV- GC tissues.**

| Category | Term | Count | PValue | Genes | Fold Enrichment | Bonferroni | Benjamini | FDR |
| --- | --- | --- | --- | --- | --- | --- | --- | --- |
| BP | GO:0009952~anterior/posterior pattern specification | 8 | <0.001 | HOXA10, HOXB3, HOXB7, HOXA6, HOXA5, HOXC10, HOXA11, HOXB5 | 18.052 | 0.001 | 0.001 | 0.001 |
| BP | GO:0048704~embryonic skeletal system morphogenesis | 5 | <0.001 | HOXB3, HOXB7, HOXA6, HOXA5, HOXB5 | 23.143 | 0.025 | 0.012 | 0.012 |
| BP | GO:0009954~proximal/distal pattern formation | 3 | 0.007 | HOXA10, HOXC10, HOXA11 | 22.564 | 0.960 | 1 | 1 |
| BP | GO:0008584~male gonad development | 4 | 0.014 | HOXA10, TFAP2C, LRRC6, HOXA11 | 7.681 | 0.998 | 1 | 1 |
| BP | GO:0030326~embryonic limb morphogenesis | 3 | 0.02 | HOXA10, HOXC10, HOXA11 | 13.54 | 0.999 | 1 | 1 |
| BP | GO:0086073~bundle of His cell-Purkinje myocyte adhesion involved in cell communication | 2 | 0.032 | DSG2, DSC2 | 60.187 | 0.999 | 1 | 1 |
| BP | GO:0001501~skeletal system development | 4 | 0.039 | HOXA10, HOXA13, HOXC10, HOXA11 | 5.276 | 0.999 | 1 | 1 |
| CC | GO:0014704~intercalated disc | 3 | 0.025 | TMEM65, DSG2, DSC2 | 12.02 | 0.955 | 1 | 1 |
| CC | GO:0016328~lateral plasma membrane | 3 | 0.034 | CLDN3, DSG2, SNTA1 | 10.213 | 0.985 | 1 | 1 |
| CC | GO:0031594~neuromuscular junction | 3 | 0.036 | CIB2, PPP1R9A, SNTA1 | 9.843 | 0.989 | 1 | 1 |
| CC | GO:0005887~integral component of plasma membrane | 14 | 0.045 | SLC22A3, VIPR1, OPN3, PODXL2, TPBG, SLC16A10, PTPRG, EFNA1, SLC6A8, CLDN3, GPC1, PLA2R1, SLC25A4, LRRC8E | 1.785 | 0.996 | 1 | 1 |
| MF | GO:0043565~sequence-specific DNA binding | 9 | 0.008 | HOXA10, FOXD1, HOXB3, HOXB7, HOXA6, HOXA13, HOXA5, HOXC10, HOXA11 | 3.087 | 0.757 | 1 | 1 |
| MF | GO:0089720~caspase binding | 2 | 0.027 | BCL2L10, NOL3 | 71.078 | 0.991 | 1 | 1 |
| MF | GO:0086083~cell adhesive protein binding involved in bundle of His cell-Purkinje myocyte communication | 2 | 0.032 | DSG2, DSC2 | 59.237 | 0.996 | 1 | 1 |
| MF | GO:0005509~calcium ion binding | 9 | 0.046 | CGREF1, F12, TNNC2, ME1, CIB2, DSG2, NOL3, NINL, DSC2 | 2.233 | 0.999 | 1 | 1 |

**Supplemental Table 5. KEGG analysis of DEGs between EBV+ vs. EBV- GC tissues.**

| Term | Count | P Value | Genes | Fold Enrichment | Bonferroni | Benjamini | FDR |
| --- | --- | --- | --- | --- | --- | --- | --- |
| Up-regulated DEGs | | | | | | | |
| hsa04612:Antigen processing and presentation | 18 | <0.001 | CD74, TAP2, TAP1, HLA-A, IFI30, CTSS, HLA-E, HLA-DMA, HLA-DMB, IFNG, CD8A, HLA-DPB1, HLA-DRA, KLRD1, HLA-DOA, LGMN, HLA-DPA1, HLA-DQB1 | 14.418 | <0.001 | <0.001 | <0.001 |
| hsa05150:Staphylococcus aureus infection | 15 | <0.001 | C1QB, C1QA, ITGB2, PTAFR, FPR3, ITGAL, ICAM1, HLA-DMA, HLA-DMB, HLA-DPB1, HLA-DRA, HLA-DOA, HLA-DPA1, C1QC, HLA-DQB1 | 16.910 | <0.001 | <0.001 | <0.001 |
| hsa05330:Allograft rejection | 13 | <0.001 | CD40, GZMB, FASLG, HLA-A, HLA-E, HLA-DMA, HLA-DMB, IFNG, HLA-DPB1, HLA-DRA, HLA-DOA, HLA-DPA1, HLA-DQB1 | 21.388 | <0.001 | <0.001 | <0.001 |
| hsa04514:Cell adhesion molecules (CAMs) | 20 | <0.001 | CD40, VCAM1, ITGB2, HLA-A, ITGAL, ICAM1, HLA-E, CD2, HLA-DMA, HLA-DMB, CD8A, HLA-DPB1, CLDN18, HLA-DRA, ITGB7, SIGLEC1, TIGIT, HLA-DOA, HLA-DPA1, HLA-DQB1 | 8.574 | <0.001 | <0.001 | <0.001 |
| hsa05332:Graft-versus-host disease | 12 | <0.001 | HLA-DMA, HLA-DMB, IFNG, HLA-DPB1, HLA-DRA, GZMB, FASLG, HLA-A, HLA-DOA, HLA-DPA1, HLA-E, HLA-DQB1 | 22.136 | <0.001 | <0.001 | <0.001 |
| hsa04940:Type I diabetes mellitus | 12 | <0.001 | HLA-DMA, HLA-DMB, IFNG, HLA-DPB1, HLA-DRA, GZMB, FASLG, HLA-A, HLA-DOA, HLA-DPA1, HLA-E, HLA-DQB1 | 17.393 | <0.001 | <0.001 | <0.001 |
| hsa05416:Viral myocarditis | 13 | <0.001 | CD40, ITGB2, HLA-A, ITGAL, ICAM1, HLA-E, HLA-DMA, HLA-DMB, HLA-DPB1, HLA-DRA, HLA-DOA, HLA-DPA1, HLA-DQB1 | 13.884 | <0.001 | <0.001 | <0.001 |
| hsa05168:Herpes simplex infection | 20 | <0.001 | CD74, TNFSF14, STAT1, STAT2, TAP2, TAP1, FASLG, HLA-A, HLA-E, HLA-DMA, HLA-DMB, IFNG, OAS2, CCL5, HLA-DPB1, HLA-DRA, JAK2, HLA-DOA, HLA-DPA1, HLA-DQB1 | 6.653 | <0.001 | <0.001 | <0.001 |
| hsa05320:Autoimmune thyroid disease | 12 | <0.001 | HLA-DMA, CD40, HLA-DMB, HLA-DPB1, HLA-DRA, GZMB, FASLG, HLA-A, HLA-DOA, HLA-DPA1, HLA-E, HLA-DQB1 | 14.048 | <0.001 | <0.001 | <0.001 |
| hsa05323:Rheumatoid arthritis | 14 | <0.001 | ITGB2, ITGAL, TNFSF13B, ICAM1, HLA-DMA, HLA-DMB, IFNG, CCL5, HLA-DPB1, HLA-DRA, ACP5, HLA-DOA, HLA-DPA1, HLA-DQB1 | 9.684 | <0.001 | <0.001 | <0.001 |
| hsa04650:Natural killer cell mediated cytotoxicity | 15 | <0.001 | FCER1G, SH2D1A, ITGB2, PIK3CD, GZMB, FASLG, ITGAL, ICAM1, ZAP70, TYROBP, IFNG, LCP2, KLRD1, CD247, HCST | 7.484 | <0.001 | <0.001 | <0.001 |
| hsa05164:Influenza A | 17 | <0.001 | STAT1, STAT2, PIK3CD, FASLG, ICAM1, CXCL10, HLA-DMA, HLA-DMB, IFNG, OAS2, CCL5, HLA-DPB1, HLA-DRA, JAK2, HLA-DOA, HLA-DPA1, HLA-DQB1 | 5.947 | <0.001 | <0.001 | <0.001 |
| hsa05310:Asthma | 9 | <0.001 | HLA-DMA, CD40, HLA-DMB, FCER1G, HLA-DPB1, HLA-DRA, HLA-DOA, HLA-DPA1, HLA-DQB1 | 18.262 | <0.001 | <0.001 | <0.001 |
| hsa05166:HTLV-I infection | 20 | <0.001 | CD40, VCAM1, ITGB2, PIK3CD, CD3G, HLA-A, ITGAL, CD3E, CD3D, ICAM1, HLA-E, HLA-DMA, HLA-DMB, IL2RA, IL2RB, HLA-DPB1, HLA-DRA, HLA-DOA, HLA-DPA1, HLA-DQB1 | 4.793 | <0.001 | <0.001 | <0.001 |
| hsa04672:Intestinal immune network for IgA production | 10 | <0.001 | HLA-DMA, CD40, HLA-DMB, HLA-DPB1, HLA-DRA, ITGB7, HLA-DOA, TNFSF13B, HLA-DPA1, HLA-DQB1 | 12.952 | <0.001 | <0.001 | <0.001 |
| Down-regulated DEGs | | | | | | | |
| hsa04146:Peroxisome | 4 | 0.005 | MPV17L, NUDT19, PXMP4, MLYCD | 10.359 | 0.301 | 0.358 | 0.358 |

**Supplemental Table 6. WGCNA analysis of all module genes.**

| Gene | Group | R | P | KME | KWI |
| --- | --- | --- | --- | --- | --- |
| GBP1 | blue | 0.88741109 | 8.80E-89 | 0.88741109 | 2.56524542 |
| IRF1 | blue | 0.87684301 | 5.53E-84 | 0.87684301 | 2.12392852 |
| LAP3 | blue | 0.86402605 | 1.00E-78 | 0.86402605 | 2.19508355 |
| FASLG | blue | 0.86009987 | 3.15E-77 | 0.86009987 | 2.22039565 |
| GZMB | blue | 0.85709098 | 4.10E-76 | 0.85709098 | 2.21486652 |
| CXCL10 | blue | 0.82585495 | 8.68E-66 | 0.82585495 | 2.38430533 |
| GZMH | blue | 0.82505834 | 1.46E-65 | 0.82505834 | 2.40558477 |
| UBE2L6 | blue | 0.82318033 | 5.06E-65 | 0.82318033 | 1.37390944 |
| CXCL9 | blue | 0.82108679 | 2.00E-64 | 0.82108679 | 1.66173243 |
| WARS | blue | 0.81936795 | 6.05E-64 | 0.81936795 | 1.75864325 |
| LAG3 | blue | 0.81701162 | 2.73E-63 | 0.81701162 | 1.29185427 |
| STAT1 | blue | 0.81120553 | 1.07E-61 | 0.81120553 | 1.31438031 |
| ETV7 | blue | 0.80830815 | 6.24E-61 | 0.80830815 | 1.57648399 |
| TAP1 | blue | 0.79802692 | 2.79E-58 | 0.79802692 | 1.62439477 |
| GBP5 | blue | 0.79488894 | 1.64E-57 | 0.79488894 | 1.55819143 |
| NKG7 | blue | 0.78615345 | 2.03E-55 | 0.78615345 | 1.83917294 |
| HAPLN3 | blue | 0.78434568 | 5.21E-55 | 0.78434568 | 0.84218315 |
| GZMA | blue | 0.77884692 | 9.26E-54 | 0.77884692 | 1.3591266 |
| CCL5 | blue | 0.77267534 | 2.13E-52 | 0.77267534 | 1.66517841 |
| CXCL11 | blue | 0.7665371 | 4.35E-51 | 0.7665371 | 1.72551493 |
| IFNG | blue | 0.76133778 | 5.17E-50 | 0.76133778 | 1.64311886 |
| FAM26F | blue | 0.75036406 | 8.18E-48 | 0.75036406 | 0.75924267 |
| GBP4 | blue | 0.74440001 | 1.12E-46 | 0.74440001 | 1.07073407 |
| KLRD1 | blue | 0.73504466 | 6.07E-45 | 0.73504466 | 0.8178286 |
| IL18BP | blue | 0.71977465 | 2.93E-42 | 0.71977465 | 0.68786199 |
| TAP2 | blue | 0.71664477 | 9.50E-42 | 0.71664477 | 0.89082797 |
| IDO1 | blue | 0.71517774 | 1.59E-41 | 0.71517774 | 0.70300682 |
| PSMB9 | blue | 0.70520025 | 6.58E-40 | 0.70520025 | 0.90775811 |
| ICAM1 | blue | 0.70185134 | 2.13E-39 | 0.70185134 | 0.37986132 |
| EPSTI1 | blue | 0.68758372 | 3.16E-37 | 0.68758372 | 0.37940049 |
| BTN3A1 | blue | 0.67568449 | 1.62E-35 | 0.67568449 | 0.40098313 |
| ZNF683 | blue | 0.64867529 | 6.88E-32 | 0.64867529 | 0.52659112 |
| RARRES3 | blue | 0.64814863 | 7.46E-32 | 0.64814863 | 0.17116238 |
| PSMB10 | blue | 0.64723288 | 9.02E-32 | 0.64723288 | 0.44173413 |
| BST2 | blue | 0.64356882 | 2.42E-31 | 0.64356882 | 0.23510405 |
| OAS2 | blue | 0.63597969 | 1.93E-30 | 0.63597969 | 0.27189534 |
| GBP2 | blue | 0.62080705 | 1.12E-28 | 0.62080705 | 0.15562249 |
| GNLY | blue | 0.61055549 | 1.47E-27 | 0.61055549 | 0.2927103 |
| SLC9A7 | blue | 0.57544958 | 6.38E-24 | 0.57544958 | 0.16641868 |
| OASL | blue | 0.55916886 | 2.02E-22 | 0.55916886 | 0.06511021 |
| DHX58 | blue | 0.52260355 | 2.82E-19 | 0.52260355 | 0.05394985 |
| JAK2 | blue | 0.5202049 | 3.57E-19 | 0.5202049 | 0.12587257 |
| LGALS9 | blue | 0.51707244 | 4.83E-19 | 0.51707244 | 0.04816082 |
| BTN3A2 | blue | 0.50342134 | 3.93E-18 | 0.50342134 | 0.13205342 |
| HLA-A | blue | 0.45603148 | 5.15E-15 | 0.45603148 | 0.0644487 |
| BIN2 | brown | 0.91574641 | 5.07E-104 | 0.91574641 | 10.3502621 |
| ITGAL | brown | 0.9048334 | 2.17E-97 | 0.9048334 | 12.3941283 |
| LILRB1 | brown | 0.9017159 | 1.21E-95 | 0.9017159 | 7.89970779 |
| CD2 | brown | 0.90132426 | 1.97E-95 | 0.90132426 | 10.0155587 |
| CD96 | brown | 0.89783436 | 1.49E-93 | 0.89783436 | 11.1209911 |
| CCR5 | brown | 0.88567046 | 1.71E-87 | 0.88567046 | 8.00610428 |
| MYO1F | brown | 0.88333817 | 2.06E-86 | 0.88333817 | 7.47058532 |
| CD3G | brown | 0.87506215 | 9.47E-83 | 0.87506215 | 9.44622188 |
| SH2D1A | brown | 0.8650651 | 1.17E-78 | 0.8650651 | 10.739807 |
| GIMAP4 | brown | 0.86144817 | 2.93E-77 | 0.86144817 | 7.79490977 |
| DOK2 | brown | 0.85526631 | 5.88E-75 | 0.85526631 | 5.87336483 |
| CD247 | brown | 0.85167943 | 1.14E-73 | 0.85167943 | 8.74014767 |
| IL2RB | brown | 0.85029682 | 3.47E-73 | 0.85029682 | 6.56712408 |
| LAIR1 | brown | 0.84911341 | 8.92E-73 | 0.84911341 | 7.06589336 |
| THEMIS | brown | 0.8445181 | 3.31E-71 | 0.8445181 | 7.11197776 |
| CD84 | brown | 0.84371122 | 6.13E-71 | 0.84371122 | 6.40200669 |
| CRTAM | brown | 0.84033051 | 8.00E-70 | 0.84033051 | 7.20293634 |
| PYHIN1 | brown | 0.84027272 | 8.29E-70 | 0.84027272 | 9.40922004 |
| CECR1 | brown | 0.83705366 | 9.03E-69 | 0.83705366 | 4.6908862 |
| UBASH3A | brown | 0.83657692 | 1.27E-68 | 0.83657692 | 8.39840701 |
| TIGIT | brown | 0.83458382 | 5.40E-68 | 0.83458382 | 7.45744189 |
| TRAT1 | brown | 0.83333341 | 1.32E-67 | 0.83333341 | 8.78597466 |
| TNFAIP8L2 | brown | 0.83265469 | 2.13E-67 | 0.83265469 | 5.68693326 |
| SLAMF8 | brown | 0.83222472 | 2.86E-67 | 0.83222472 | 6.85246455 |
| CD3D | brown | 0.82688632 | 1.20E-65 | 0.82688632 | 6.88086681 |
| AOAH | brown | 0.82297807 | 1.69E-64 | 0.82297807 | 5.21234999 |
| FAM78A | brown | 0.82174428 | 3.83E-64 | 0.82174428 | 7.83832633 |
| CYBB | brown | 0.8210089 | 6.20E-64 | 0.8210089 | 6.27349545 |
| DOCK10 | brown | 0.82028203 | 9.94E-64 | 0.82028203 | 5.55778198 |
| CD3E | brown | 0.81907526 | 2.18E-63 | 0.81907526 | 7.74794041 |
| ARHGAP9 | brown | 0.81848258 | 3.18E-63 | 0.81848258 | 7.98494103 |
| GNGT2 | brown | 0.81504728 | 2.90E-62 | 0.81504728 | 4.72322363 |
| NFAM1 | brown | 0.80546126 | 1.12E-59 | 0.80546126 | 3.8771594 |
| CMKLR1 | brown | 0.80379057 | 3.02E-59 | 0.80379057 | 5.32180412 |
| GPR171 | brown | 0.80056188 | 2.03E-58 | 0.80056188 | 4.68296011 |
| C1QC | brown | 0.79918589 | 4.49E-58 | 0.79918589 | 5.85009313 |
| C1QB | brown | 0.79827624 | 7.54E-58 | 0.79827624 | 6.0855015 |
| CXCR6 | brown | 0.79711589 | 1.46E-57 | 0.79711589 | 4.00019258 |
| HLA-DPA1 | brown | 0.796173 | 2.49E-57 | 0.796173 | 4.55434574 |
| PSTPIP1 | brown | 0.79578412 | 3.07E-57 | 0.79578412 | 5.60509154 |
| LCP2 | brown | 0.79520617 | 4.23E-57 | 0.79520617 | 4.17956022 |
| GMFG | brown | 0.79050408 | 5.91E-56 | 0.79050408 | 4.6552641 |
| PREX1 | brown | 0.78916251 | 1.23E-55 | 0.78916251 | 3.46982314 |
| C1QA | brown | 0.7861411 | 6.34E-55 | 0.7861411 | 4.90468775 |
| NCKAP1L | brown | 0.78544546 | 9.14E-55 | 0.78544546 | 4.55879227 |
| CD8A | brown | 0.78534226 | 9.56E-55 | 0.78534226 | 4.74193794 |
| P2RY13 | brown | 0.78359542 | 2.42E-54 | 0.78359542 | 3.67205366 |
| HAVCR2 | brown | 0.7831442 | 3.04E-54 | 0.7831442 | 6.13388872 |
| HLA-DPB1 | brown | 0.78304039 | 3.18E-54 | 0.78304039 | 4.49244355 |
| FYB | brown | 0.77971258 | 1.83E-53 | 0.77971258 | 3.20885213 |
| PIK3CD | brown | 0.77673464 | 8.51E-53 | 0.77673464 | 6.38544014 |
| MS4A6A | brown | 0.77597579 | 1.24E-52 | 0.77597579 | 5.1982177 |
| GIMAP6 | brown | 0.77481419 | 2.23E-52 | 0.77481419 | 4.21553495 |
| CD300LF | brown | 0.77385178 | 3.60E-52 | 0.77385178 | 5.32400617 |
| GZMK | brown | 0.77327957 | 4.76E-52 | 0.77327957 | 4.94111508 |
| AIF1 | brown | 0.77197821 | 9.07E-52 | 0.77197821 | 4.66364553 |
| IGSF6 | brown | 0.76899706 | 3.98E-51 | 0.76899706 | 3.71118757 |
| NLRC3 | brown | 0.75714806 | 1.18E-48 | 0.75714806 | 6.42072069 |
| CD74 | brown | 0.75535777 | 2.69E-48 | 0.75535777 | 3.85163668 |
| HLA-DRA | brown | 0.75526489 | 2.78E-48 | 0.75526489 | 3.88219694 |
| SLAMF7 | brown | 0.75376768 | 5.47E-48 | 0.75376768 | 2.74412875 |
| HCK | brown | 0.75067397 | 2.22E-47 | 0.75067397 | 3.38187982 |
| TNFSF13B | brown | 0.74636258 | 1.52E-46 | 0.74636258 | 1.98776825 |
| ITGB2 | brown | 0.74406182 | 4.14E-46 | 0.74406182 | 3.02350148 |
| LST1 | brown | 0.74352882 | 5.16E-46 | 0.74352882 | 3.38426084 |
| HCST | brown | 0.74149055 | 1.24E-45 | 0.74149055 | 3.68046526 |
| AMICA1 | brown | 0.74104399 | 1.48E-45 | 0.74104399 | 2.22167472 |
| LILRB4 | brown | 0.74019412 | 2.11E-45 | 0.74019412 | 3.98759247 |
| TRIM22 | brown | 0.7393951 | 2.94E-45 | 0.7393951 | 2.13272955 |
| KLHL6 | brown | 0.7331716 | 4.06E-44 | 0.7331716 | 3.71045048 |
| APOL3 | brown | 0.73081199 | 1.07E-43 | 0.73081199 | 1.93558484 |
| ITGB7 | brown | 0.72938237 | 1.89E-43 | 0.72938237 | 2.00008887 |
| IL2RA | brown | 0.72799695 | 3.29E-43 | 0.72799695 | 2.91491577 |
| GZMM | brown | 0.72755449 | 3.88E-43 | 0.72755449 | 4.05312775 |
| GIMAP2 | brown | 0.72100099 | 5.33E-42 | 0.72100099 | 1.54483419 |
| FPR3 | brown | 0.72090384 | 5.45E-42 | 0.72090384 | 4.88937981 |
| HLA-DMB | brown | 0.71788397 | 1.76E-41 | 0.71788397 | 3.16284217 |
| TLR8 | brown | 0.71622537 | 3.30E-41 | 0.71622537 | 4.12571952 |
| LAX1 | brown | 0.71534977 | 4.55E-41 | 0.71534977 | 3.15617436 |
| MS4A7 | brown | 0.7150464 | 5.03E-41 | 0.7150464 | 3.1292946 |
| MIAT | brown | 0.71447367 | 6.16E-41 | 0.71447367 | 2.1580726 |
| ACAP1 | brown | 0.71338846 | 9.19E-41 | 0.71338846 | 6.44035443 |
| GIMAP7 | brown | 0.71186084 | 1.62E-40 | 0.71186084 | 4.10750454 |
| PLA2G7 | brown | 0.70623501 | 1.32E-39 | 0.70623501 | 3.80410274 |
| SIGLEC1 | brown | 0.70572885 | 1.56E-39 | 0.70572885 | 2.05804083 |
| VCAM1 | brown | 0.70529543 | 1.80E-39 | 0.70529543 | 1.43504544 |
| HLA-DOA | brown | 0.70435855 | 2.49E-39 | 0.70435855 | 1.70520284 |
| TYROBP | brown | 0.70253851 | 4.77E-39 | 0.70253851 | 3.52133217 |
| GIMAP8 | brown | 0.70087183 | 8.59E-39 | 0.70087183 | 2.74337023 |

**Supplemental Table 7. Key hub genes of |kME| ≥ 0.7 in each respective module.**

| Gene | Group | R | P | KME | KWI |
| --- | --- | --- | --- | --- | --- |
| GBP1 | blue | 0.88741109 | 8.80E-89 | 0.88741109 | 2.56524542 |
| IRF1 | blue | 0.87684301 | 5.53E-84 | 0.87684301 | 2.12392852 |
| LAP3 | blue | 0.86402605 | 1.00E-78 | 0.86402605 | 2.19508355 |
| FASLG | blue | 0.86009987 | 3.15E-77 | 0.86009987 | 2.22039565 |
| GZMB | blue | 0.85709098 | 4.10E-76 | 0.85709098 | 2.21486652 |
| CXCL10 | blue | 0.82585495 | 8.68E-66 | 0.82585495 | 2.38430533 |
| GZMH | blue | 0.82505834 | 1.46E-65 | 0.82505834 | 2.40558477 |
| UBE2L6 | blue | 0.82318033 | 5.06E-65 | 0.82318033 | 1.37390944 |
| CXCL9 | blue | 0.82108679 | 2.00E-64 | 0.82108679 | 1.66173243 |
| WARS | blue | 0.81936795 | 6.05E-64 | 0.81936795 | 1.75864325 |
| LAG3 | blue | 0.81701162 | 2.73E-63 | 0.81701162 | 1.29185427 |
| STAT1 | blue | 0.81120553 | 1.07E-61 | 0.81120553 | 1.31438031 |
| ETV7 | blue | 0.80830815 | 6.24E-61 | 0.80830815 | 1.57648399 |
| TAP1 | blue | 0.79802692 | 2.79E-58 | 0.79802692 | 1.62439477 |
| GBP5 | blue | 0.79488894 | 1.64E-57 | 0.79488894 | 1.55819143 |
| NKG7 | blue | 0.78615345 | 2.03E-55 | 0.78615345 | 1.83917294 |
| HAPLN3 | blue | 0.78434568 | 5.21E-55 | 0.78434568 | 0.84218315 |
| GZMA | blue | 0.77884692 | 9.26E-54 | 0.77884692 | 1.3591266 |
| CCL5 | blue | 0.77267534 | 2.13E-52 | 0.77267534 | 1.66517841 |
| CXCL11 | blue | 0.7665371 | 4.35E-51 | 0.7665371 | 1.72551493 |
| IFNG | blue | 0.76133778 | 5.17E-50 | 0.76133778 | 1.64311886 |
| FAM26F | blue | 0.75036406 | 8.18E-48 | 0.75036406 | 0.75924267 |
| GBP4 | blue | 0.74440001 | 1.12E-46 | 0.74440001 | 1.07073407 |
| KLRD1 | blue | 0.73504466 | 6.07E-45 | 0.73504466 | 0.8178286 |
| IL18BP | blue | 0.71977465 | 2.93E-42 | 0.71977465 | 0.68786199 |
| TAP2 | blue | 0.71664477 | 9.50E-42 | 0.71664477 | 0.89082797 |
| IDO1 | blue | 0.71517774 | 1.59E-41 | 0.71517774 | 0.70300682 |
| PSMB9 | blue | 0.70520025 | 6.58E-40 | 0.70520025 | 0.90775811 |
| ICAM1 | blue | 0.70185134 | 2.13E-39 | 0.70185134 | 0.37986132 |
| BIN2 | brown | 0.91574641 | 5.07E-104 | 0.91574641 | 10.3502621 |
| ITGAL | brown | 0.9048334 | 2.17E-97 | 0.9048334 | 12.3941283 |
| LILRB1 | brown | 0.9017159 | 1.21E-95 | 0.9017159 | 7.89970779 |
| CD2 | brown | 0.90132426 | 1.97E-95 | 0.90132426 | 10.0155587 |
| CD96 | brown | 0.89783436 | 1.49E-93 | 0.89783436 | 11.1209911 |
| CCR5 | brown | 0.88567046 | 1.71E-87 | 0.88567046 | 8.00610428 |
| MYO1F | brown | 0.88333817 | 2.06E-86 | 0.88333817 | 7.47058532 |
| CD3G | brown | 0.87506215 | 9.47E-83 | 0.87506215 | 9.44622188 |
| SH2D1A | brown | 0.8650651 | 1.17E-78 | 0.8650651 | 10.739807 |
| GIMAP4 | brown | 0.86144817 | 2.93E-77 | 0.86144817 | 7.79490977 |
| DOK2 | brown | 0.85526631 | 5.88E-75 | 0.85526631 | 5.87336483 |
| CD247 | brown | 0.85167943 | 1.14E-73 | 0.85167943 | 8.74014767 |
| IL2RB | brown | 0.85029682 | 3.47E-73 | 0.85029682 | 6.56712408 |
| LAIR1 | brown | 0.84911341 | 8.92E-73 | 0.84911341 | 7.06589336 |
| THEMIS | brown | 0.8445181 | 3.31E-71 | 0.8445181 | 7.11197776 |
| CD84 | brown | 0.84371122 | 6.13E-71 | 0.84371122 | 6.40200669 |
| CRTAM | brown | 0.84033051 | 8.00E-70 | 0.84033051 | 7.20293634 |
| PYHIN1 | brown | 0.84027272 | 8.29E-70 | 0.84027272 | 9.40922004 |
| CECR1 | brown | 0.83705366 | 9.03E-69 | 0.83705366 | 4.6908862 |
| UBASH3A | brown | 0.83657692 | 1.27E-68 | 0.83657692 | 8.39840701 |
| TIGIT | brown | 0.83458382 | 5.40E-68 | 0.83458382 | 7.45744189 |
| TRAT1 | brown | 0.83333341 | 1.32E-67 | 0.83333341 | 8.78597466 |
| TNFAIP8L2 | brown | 0.83265469 | 2.13E-67 | 0.83265469 | 5.68693326 |
| SLAMF8 | brown | 0.83222472 | 2.86E-67 | 0.83222472 | 6.85246455 |
| CD3D | brown | 0.82688632 | 1.20E-65 | 0.82688632 | 6.88086681 |
| AOAH | brown | 0.82297807 | 1.69E-64 | 0.82297807 | 5.21234999 |
| FAM78A | brown | 0.82174428 | 3.83E-64 | 0.82174428 | 7.83832633 |
| CYBB | brown | 0.8210089 | 6.20E-64 | 0.8210089 | 6.27349545 |
| DOCK10 | brown | 0.82028203 | 9.94E-64 | 0.82028203 | 5.55778198 |
| CD3E | brown | 0.81907526 | 2.18E-63 | 0.81907526 | 7.74794041 |
| ARHGAP9 | brown | 0.81848258 | 3.18E-63 | 0.81848258 | 7.98494103 |
| GNGT2 | brown | 0.81504728 | 2.90E-62 | 0.81504728 | 4.72322363 |
| NFAM1 | brown | 0.80546126 | 1.12E-59 | 0.80546126 | 3.8771594 |
| CMKLR1 | brown | 0.80379057 | 3.02E-59 | 0.80379057 | 5.32180412 |
| GPR171 | brown | 0.80056188 | 2.03E-58 | 0.80056188 | 4.68296011 |
| C1QC | brown | 0.79918589 | 4.49E-58 | 0.79918589 | 5.85009313 |
| C1QB | brown | 0.79827624 | 7.54E-58 | 0.79827624 | 6.0855015 |
| CXCR6 | brown | 0.79711589 | 1.46E-57 | 0.79711589 | 4.00019258 |
| HLA-DPA1 | brown | 0.796173 | 2.49E-57 | 0.796173 | 4.55434574 |
| PSTPIP1 | brown | 0.79578412 | 3.07E-57 | 0.79578412 | 5.60509154 |
| LCP2 | brown | 0.79520617 | 4.23E-57 | 0.79520617 | 4.17956022 |
| GMFG | brown | 0.79050408 | 5.91E-56 | 0.79050408 | 4.6552641 |
| PREX1 | brown | 0.78916251 | 1.23E-55 | 0.78916251 | 3.46982314 |
| C1QA | brown | 0.7861411 | 6.34E-55 | 0.7861411 | 4.90468775 |
| NCKAP1L | brown | 0.78544546 | 9.14E-55 | 0.78544546 | 4.55879227 |
| CD8A | brown | 0.78534226 | 9.56E-55 | 0.78534226 | 4.74193794 |
| P2RY13 | brown | 0.78359542 | 2.42E-54 | 0.78359542 | 3.67205366 |
| HAVCR2 | brown | 0.7831442 | 3.04E-54 | 0.7831442 | 6.13388872 |
| HLA-DPB1 | brown | 0.78304039 | 3.18E-54 | 0.78304039 | 4.49244355 |
| FYB | brown | 0.77971258 | 1.83E-53 | 0.77971258 | 3.20885213 |
| PIK3CD | brown | 0.77673464 | 8.51E-53 | 0.77673464 | 6.38544014 |
| MS4A6A | brown | 0.77597579 | 1.24E-52 | 0.77597579 | 5.1982177 |
| GIMAP6 | brown | 0.77481419 | 2.23E-52 | 0.77481419 | 4.21553495 |
| CD300LF | brown | 0.77385178 | 3.60E-52 | 0.77385178 | 5.32400617 |
| GZMK | brown | 0.77327957 | 4.76E-52 | 0.77327957 | 4.94111508 |
| AIF1 | brown | 0.77197821 | 9.07E-52 | 0.77197821 | 4.66364553 |
| IGSF6 | brown | 0.76899706 | 3.98E-51 | 0.76899706 | 3.71118757 |
| NLRC3 | brown | 0.75714806 | 1.18E-48 | 0.75714806 | 6.42072069 |
| CD74 | brown | 0.75535777 | 2.69E-48 | 0.75535777 | 3.85163668 |
| HLA-DRA | brown | 0.75526489 | 2.78E-48 | 0.75526489 | 3.88219694 |
| SLAMF7 | brown | 0.75376768 | 5.47E-48 | 0.75376768 | 2.74412875 |
| HCK | brown | 0.75067397 | 2.22E-47 | 0.75067397 | 3.38187982 |
| TNFSF13B | brown | 0.74636258 | 1.52E-46 | 0.74636258 | 1.98776825 |
| ITGB2 | brown | 0.74406182 | 4.14E-46 | 0.74406182 | 3.02350148 |
| LST1 | brown | 0.74352882 | 5.16E-46 | 0.74352882 | 3.38426084 |
| HCST | brown | 0.74149055 | 1.24E-45 | 0.74149055 | 3.68046526 |
| AMICA1 | brown | 0.74104399 | 1.48E-45 | 0.74104399 | 2.22167472 |
| LILRB4 | brown | 0.74019412 | 2.11E-45 | 0.74019412 | 3.98759247 |
| TRIM22 | brown | 0.7393951 | 2.94E-45 | 0.7393951 | 2.13272955 |
| KLHL6 | brown | 0.7331716 | 4.06E-44 | 0.7331716 | 3.71045048 |
| APOL3 | brown | 0.73081199 | 1.07E-43 | 0.73081199 | 1.93558484 |
| ITGB7 | brown | 0.72938237 | 1.89E-43 | 0.72938237 | 2.00008887 |
| IL2RA | brown | 0.72799695 | 3.29E-43 | 0.72799695 | 2.91491577 |
| GZMM | brown | 0.72755449 | 3.88E-43 | 0.72755449 | 4.05312775 |
| GIMAP2 | brown | 0.72100099 | 5.33E-42 | 0.72100099 | 1.54483419 |
| FPR3 | brown | 0.72090384 | 5.45E-42 | 0.72090384 | 4.88937981 |
| HLA-DMB | brown | 0.71788397 | 1.76E-41 | 0.71788397 | 3.16284217 |
| TLR8 | brown | 0.71622537 | 3.30E-41 | 0.71622537 | 4.12571952 |
| LAX1 | brown | 0.71534977 | 4.55E-41 | 0.71534977 | 3.15617436 |
| MS4A7 | brown | 0.7150464 | 5.03E-41 | 0.7150464 | 3.1292946 |
| MIAT | brown | 0.71447367 | 6.16E-41 | 0.71447367 | 2.1580726 |
| ACAP1 | brown | 0.71338846 | 9.19E-41 | 0.71338846 | 6.44035443 |
| GIMAP7 | brown | 0.71186084 | 1.62E-40 | 0.71186084 | 4.10750454 |
| PLA2G7 | brown | 0.70623501 | 1.32E-39 | 0.70623501 | 3.80410274 |
| SIGLEC1 | brown | 0.70572885 | 1.56E-39 | 0.70572885 | 2.05804083 |
| VCAM1 | brown | 0.70529543 | 1.80E-39 | 0.70529543 | 1.43504544 |
| HLA-DOA | brown | 0.70435855 | 2.49E-39 | 0.70435855 | 1.70520284 |
| TYROBP | brown | 0.70253851 | 4.77E-39 | 0.70253851 | 3.52133217 |
| GIMAP8 | brown | 0.70087183 | 8.59E-39 | 0.70087183 | 2.74337023 |

**Supplemental Table 8: Pathway enrichment analysis of each MCODE component.**

| MCODE | GO | Description | Log10(P) |
| --- | --- | --- | --- |
| MCODE_1 | R-HSA-202433 | Generation of second messenger molecules | -24.2 |
| MCODE_1 | hsa04658 | Th1 and Th2 cell differentiation | -22.9 |
| MCODE_1 | hsa04659 | Th17 cell differentiation | -22.2 |
| MCODE_2 | R-HSA-418594 | G alpha (i) signalling events | -15.9 |
| MCODE_2 | R-HSA-373076 | Class A/1 (Rhodopsin-like receptors) | -15.7 |
| MCODE_2 | R-HSA-380108 | Chemokine receptors bind chemokines | -14.9 |
| MCODE_3 | GO:0045123 | cellular extravasation | -11.5 |
| MCODE_3 | GO:0007159 | leukocyte cell-cell adhesion | -11.0 |
| MCODE_3 | R-HSA-216083 | Integrin cell surface interactions | -10.2 |
| MCODE_4 | GO:0019886 | antigen processing and presentation of exogenous peptide antigen via MHC class II | -9.1 |
| MCODE_4 | GO:0002495 | antigen processing and presentation of peptide antigen via MHC class II | -9.0 |
| MCODE_4 | GO:0002504 | antigen processing and presentation of peptide or polysaccharide antigen via MHC class II | -8.9 |
